# Persistent neuropsychiatric symptoms after COVID-19: a systematic review and meta-analysis

**DOI:** 10.1093/braincomms/fcab297

**Published:** 2021-12-17

**Authors:** James B. Badenoch, Emma R. Rengasamy, Cameron Watson, Katrin Jansen, Stuti Chakraborty, Ritika D. Sundaram, Danish Hafeez, Ella Burchill, Aman Saini, Lucretia Thomas, Benjamin Cross, Camille K. Hunt, Isabella Conti, Sylvia Ralovska, Zain Hussain, Matthew Butler, Thomas A. Pollak, Ivan Koychev, Benedict D. Michael, Heinz Holling, Timothy R. Nicholson, Jonathan P. Rogers, Alasdair G. Rooney

**Affiliations:** Barts Health NHS Trust, London, UK; Queen Mary University of London, London, UK; Department of Public Health and Primary Care, University of Cambridge, Cambridge, UK; Barts Health NHS Trust, London, UK; Preventive Neurology Unit, Wolfson Institute of Preventive Medicine, Queen Mary University of London, London, UK; Department of Psychology, University of Münster, Münster, Germany; Department of Physical Medicine and Rehabilitation, Christian Medical College and Hospital, Vellore, India; Occupational Therapy Unit, Department of Physical Medicine and Rehabilitation, Christian Medical College and Hospital, Vellore, India; School of Medicine, University of Glasgow, Glasgow, UK; Homerton University Hospitals Foundation Trust, London, UK; Faculty of Medicine and Life Sciences, King’s College London, London, UK; Medical School, University College London, London, UK; College of Medical and Dental Sciences, University of Birmingham, Birmingham, UK; East Lancashire Hospitals NHS Trust, Lancashire, UK; Warwick Medical School, Coventry, UK; School of Medicine, Dentistry and Biomedical Sciences, Queen’s University Belfast, Belfast, UK; Sofia University ‘St Kliment Ohridski’, bul, ‘Tsar Osvoboditel’ 15, Sofia, Bulgaria; Edinburgh Medical School, College of Medicine and Veterinary Medicine, University of Edinburgh, UK; School of Medicine, University of Dundee, Dundee, UK; Institute of Psychiatry, Psychology and Neuroscience, King’s College London, London, UK; Department of Psychosis Studies, Institute of Psychiatry, Psychology and Neuroscience, King’s College London, London, UK; Department of Psychiatry, University of Oxford, Oxford, UK; Department of Psychological Medicine, Oxford University Hospitals NHS Foundation Trust, Oxford, UK; Department of Neurology, The Walton Centre NHS Foundation Trust, Liverpool, UK; National Institute for Health Research Health Protection Research Unit in Emerging and Zoonotic Infection, University of Liverpool, Liverpool, UK; Clinical Infection Microbiology and Immunology, Institute of Infection, Veterinary, and Zoological Science, University of Liverpool, Liverpool, UK; Department of Psychology, University of Münster, Münster, Germany; Section of Cognitive Neuropsychiatry, Institute of Psychiatry, Psychology and Neuroscience, King’s College London, London, UK; Division of Psychiatry, University College London, London, UK; South London and Maudsley NHS Foundation Trust, London, UK; Centre for Clinical Brain Sciences, The University of Edinburgh, Edinburgh, UK

**Keywords:** COVID-19, neuropsychiatry, Long COVID, post-acute sequelae of COVID-19, chronic COVID syndrome

## Abstract

The nature and extent of persistent neuropsychiatric symptoms after COVID-19 are not established. To help inform mental health service planning in the pandemic recovery phase, we systematically determined the prevalence of neuropsychiatric symptoms in survivors of COVID-19. For this pre-registered systematic review and meta-analysis (PROSPERO ID CRD42021239750), we searched MEDLINE, EMBASE, CINAHL and PsycINFO to 20 February 2021, plus our own curated database. We included peer-reviewed studies reporting neuropsychiatric symptoms at post-acute or later time-points after COVID-19 infection and in control groups where available. For each study, a minimum of two authors extracted summary data. For each symptom, we calculated a pooled prevalence using generalized linear mixed models. Heterogeneity was measured with *I*^2^. Subgroup analyses were conducted for COVID-19 hospitalization, severity and duration of follow-up. From 2844 unique titles, we included 51 studies (*n *= 18 917 patients). The mean duration of follow-up after COVID-19 was 77 days (range 14–182 days). Study quality was most commonly moderate. The most prevalent neuropsychiatric symptom was sleep disturbance [pooled prevalence = 27.4% (95% confidence interval 21.4–34.4%)], followed by fatigue [24.4% (17.5–32.9%)], objective cognitive impairment [20.2% (10.3–35.7%)], anxiety [19.1% (13.3–26.8%)] and post-traumatic stress [15.7% (9.9–24.1%)]. Only two studies reported symptoms in control groups, both reporting higher frequencies in COVID-19 survivors versus controls. Between-study heterogeneity was high (*I*^2 ^= 79.6–98.6%). There was little or no evidence of differential symptom prevalence based on hospitalization status, severity or follow-up duration. Neuropsychiatric symptoms are common and persistent after recovery from COVID-19. The literature on longer-term consequences is still maturing but indicates a particularly high prevalence of insomnia, fatigue, cognitive impairment and anxiety disorders in the first 6 months after infection.

## Teaser

In a systematic review and meta-analysis, Badenoch *et al.* report frequent neuropsychiatric symptoms persisting up to 6 months after COVID-19, including insomnia, fatigue, anxiety, post-traumatic symptoms, cognitive impairment and mood disorder. Health services should plan for a high requirement for multidisciplinary services (including neurological, neuropsychiatric and psychological management) after COVID-19.

## Graphical Abstract

**Graphical Abstract:**
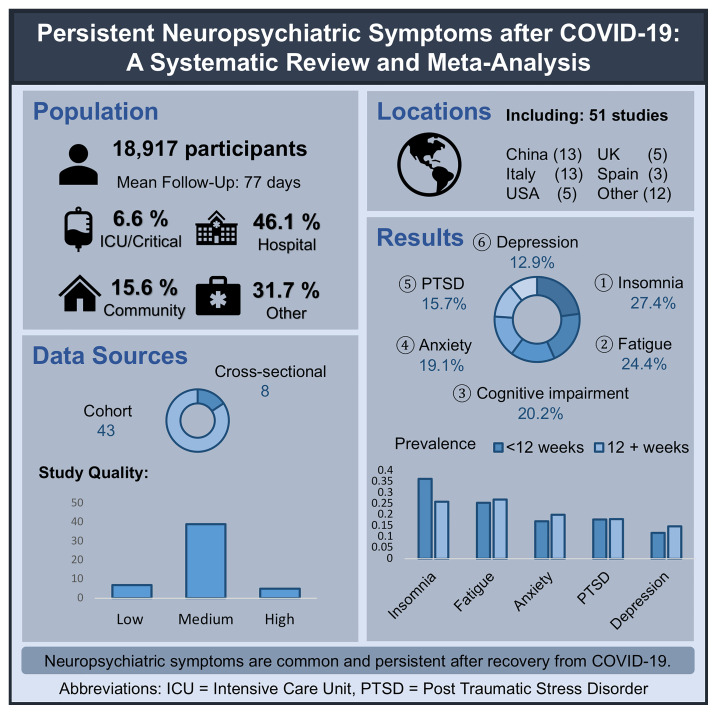

## Introduction

Early in the COVID-19 pandemic, neuropsychiatric symptoms were identified as a prominent feature of coronavirus outbreaks.^1,2^ Analyses subsequently confirmed many neuropsychiatric manifestations of acute infection with severe acute respiratory syndrome coronavirus 2 (SARS-CoV-2), with non-specific symptoms such as fatigue and headache, the most commonly studied and reported in the early literature.^3^ Studies assessing the prevalence of depression, anxiety and post-traumatic stress in acute COVID-19 suggested specific psychiatric morbidity. The degree of persistence of neuropsychiatric symptoms in the post-acute and chronic phases after infection, however, remained far from clear.

Persistent symptoms after COVID-19 illness have been called ‘Long COVID’.^4–7^ The point of onset of Long COVID is imprecisely defined and has been proposed to range from 3 to 12 weeks after infection.^8^ Separately, National Institute for Health and Clinical Excellence (NICE) guidelines from the UK conceptualize symptoms persisting between 4 and 12 weeks after infection as ‘ongoing symptomatic COVID-19’, with ‘post-COVID-19 syndrome’ thereafter.^9^ However it is defined, persistent symptoms after COVID-19 are considered to be multi-system in nature with most likely several distinct pathological mechanisms.^10–12^ These uncertainties of definitions, terminology and mechanism reflect the early stage of our knowledge about persisting symptoms after COVID-19, and in particular the lack of systematized descriptions of different components of the syndrome.

Emerging reports suggest a high frequency of neuropsychiatric symptoms after infection with COVID-19. These reports emphasize fatigue, cognitive dysfunction and sleep disorders, with increased rates of newly diagnosed mood or anxiety disorders, and dementia.^13–15^ Whether and how these neuropsychiatric sequelae are influenced by the severity of initial illness, or by the duration since COVID-19, is not known. The answers are however important, both for our theoretical understanding of the extent and scope of COVID-19 sequelae, and to ensure sufficient provision of clinical services for COVID-19 survivors.^8,16,17^ Previous analyses either did not focus specifically on these outcomes^18^ or have been superseded by the rapid growth in research.^19^

To further characterize the consequences of infection and help inform service planning, therefore, we aimed to estimate the prevalence of persistent neuropsychiatric symptoms in survivors of COVID-19. In secondary analyses, we aimed to identify predictors of symptom prevalence. We hypothesized that persistent neuropsychiatric symptoms would be common among survivors of COVID-19, particularly in those with a more severe form of the illness (i.e. those that have required hospitalization or intensive care), and would lessen in frequency as time passed after infection.

## Materials and methods

We conducted a systematic review and meta-analysis based on a pre-registered protocol (PROSPERO ID CRD42021239750) reported according to PRISMA guidelines.^20^ A detailed list of author contributions is provided (Supplementary Table 1).

### Search strategy

We searched Ovid MEDLINE^®^ and Epub Ahead of Print, In-Process and Other Non-Indexed Citations and Daily, EMBASE (via Ovid), APA PsycInfo (via OVID) and CINAHL (via EBSCO) from 1 January 2020 to 20 February 2021. We adapted a previously published, librarian-designed search strategy for post-acute, persisting or Long COVID.^21^ To maximize sensitivity, our search strategy (Supplementary Methods) did not specify neuropsychiatric terms. We further examined our weekly curated database of COVID-19 neurology and neuropsychiatry research^22^ for any papers that were missed by the search strategy and screened the reference lists of relevant systematic reviews published at the time of our primary search.^18,19^

### Eligibility criteria

We included any observational study reporting persistent neuropsychiatric symptoms in adults (aged 18+ years), with a history of polymerase chain reaction (PCR)-confirmed or clinically suspected SARS-CoV-2 infection. We defined the ‘persistence’ of symptoms differently for hospitalized and community-based samples. In hospitalized samples, we considered persistent symptoms as those present after hospital discharge, because discharged individuals are generally beyond the acute illness phase. In community-based samples, which lacked a discharge date, we considered persistent symptoms as those still present at least 4 weeks after the onset of symptoms or a positive PCR test.

We adopted a definition of ‘neuropsychiatric’ symptoms proposed by patient-led research in this area.^15^ We studied: affective symptoms (specifically anxiety and panic attacks, depression and mania); hallucinations; sleep disturbance; objectively reported cognitive impairment (i.e. through standardized cognitive tests); subjective cognitive impairment (such as the patient report of ‘brain fog’ or other lay terms); sensorimotor symptoms (such as paraesthesia, numbness or weakness of specific body parts); dizziness and vertigo; headache; changes in speech or language and changes in taste or smell. We added fatigue, which in our experience is commonly encountered in Long COVID clinics, and post-traumatic stress disorder or symptoms (PTSD/PTSS), which are frequently reported after COVID-19 (Box 1).

Box 1 Neuropsychiatric symptoms recorded in this review

Anxiety

Depression

Mania

Sensorimotor*

Dizziness/vertigo

Sleep changes

Objectively measured cognitive dysfunction

Subjectively reported cognitive dysfunction

Headache

Reduced taste

Reduced smell

Speech or language difficulty

Hallucinations

Fatigue

Panic

Post-traumatic stress disorder/symptoms

*Paraesthesia, numbness or weakness of specific body parts.

We excluded studies which did not report original data; where patients were not infected (or presumed infected) with SARS-CoV-2; had fewer than 10 COVID-19 patients; reported no post-discharge data (hospitalized samples) or no time-points longer than 4 weeks post-diagnosis (community samples); did not report any of the neuropsychiatric symptoms listed above; were not in the English language; were preclinical (animal/laboratory-based); or had not been peer-reviewed. In addition and considering our main aim, studies were only eligible if their design permitted, in our opinion, a reasonably generalizable estimation of point prevalence to the wider population. On this basis, for example, we excluded studies in which participants were eligible solely because of predetermined characteristics (e.g. the presence of neurological symptoms), had been discharged to ongoing inpatient rehabilitation for persisting symptoms, or were primarily drawn from statistically enriched samples such as support groups designed for people with persisting symptoms or those drawn from samples exclusively with a specific pre-existing condition. Senior authors (A.G.R./J.P.R.) discussed and agreed decisions about eligibility taken on this basis (see also the Results section).

### Screening and data extraction

Screening of titles, abstracts and the full text was conducted by a minimum of two authors each blinded to the other’s ratings using Rayyan (www.rayyan.ai). Lead authors (A.G.R./J.P.R./J.B.B.) resolved assessment discrepancies. For each eligible study, data were extracted to a customized spreadsheet by one reviewer, then checked for accuracy by a second reviewer.

We aimed to extract all usable data for primary and secondary analyses. For instance, if a study reported data on the total population plus a breakdown of the same data for individual subgroups, we extracted each group (total population, Subgroup 1, Subgroup 2, etc.) separately to the database. In the primary analysis, we only included data from the total population of each study. In order to qualify for a subgroup analysis, we took the conservative position that a study had to report extractable data on a completely homogeneous subgroup (e.g. we would not label studies reporting a combined 95% community and 5% hospitalized patients as ‘community’ samples; such a study would instead be excluded from the ‘hospital versus community’ secondary analysis). Where a study reported multiple time-points, we included only the longest follow-up time-point. Where data were presented in a way which did not meet our purposes, we contacted study investigators to request clarification. The quality of each study was graded using the Newcastle–Ottawa Scale by a minimum of two authors working independently. A full list of data fields and outcomes extracted is presented in Supplementary Table 2.

### Outcomes

The primary outcome was the pooled prevalence of each neuropsychiatric symptom, using estimates of point prevalence where available. We recorded symptoms however defined or measured by study investigators, including on the basis of patient self-report, clinical interview, case-note review or rating scale. Where rating scales were used to quantify symptoms, we noted the specific scale and threshold applied. Where symptoms were characterized as a dichotomous variable (present/absent), including for patients scoring above a rating scale threshold, we recorded the relative frequency (‘*n* affected in study’ divided by ‘*n* infected with SARS-CoV-2’). Where symptoms were reported as continuous or ordinal variables (e.g. using rating scales) *and* where such data were available for both COVID-19 patients and a control group, we intended to calculate and then pool the standardized mean differences. Studies reporting continuous or ordinal variables in COVID-19 populations only were summarized narratively in a table.

### Statistical analysis

#### Primary analysis

We conducted the primary analysis on every neuropsychiatric symptom reported by three or more studies. We pooled results based on random-effects meta-analysis, using the metafor package^23^ in R version 4.0.2 to calculate generalized linear mixed models for each prevalence outcome,^24,25^ before using the inverse variance method with the Freeman–Tukey double arcsine transformation as a comparative sensitivity analysis.^26^ We assessed between-study heterogeneity using the *I*² statistic. For interpretation, we reported forest plots with 95% confidence intervals (CIs).

Around one-fifth of eligible studies reported multiple types of cognitive dysfunction. *Post hoc* we separated cognitive dysfunction into ‘objective’ and ‘subjective’ dysfunction. We defined objective cognitive dysfunction as revealed by a cognitive assessment screening tool (e.g. Mini-Mental State Examination, Montreal Cognitive Assessment or similar). All other forms of cognitive dysfunction (such as patient self-report of memory problems, ‘brain fog’ or similar) were classed as subjective. A small number of studies reported >1 symptom of subjective cognitive dysfunction (for example, self-reported ‘memory disorder’ and ‘concentration disorder’)^27^ and in such cases, we included the subjective cognitive symptom with the highest prevalence.

#### Secondary analyses

*A priori* we intended to conduct secondary analyses examining for differences in neuropsychiatric symptom prevalence between (i) COVID-19 patients and control groups, (ii) COVID-19 patients whose diagnosis was PCR-confirmed and those in whom it was not, (iii) hospitalized and community samples and (iv) different time-points following a positive test for SARS-CoV-2 (specifying <12 weeks versus 12 or more weeks to align with a key time-point in NICE guidance for post-COVID-19 syndrome.^9^ Following data extraction, we discovered that analyses (i) and (ii) could not be run owing to a dearth of studies with control groups or non-PCR-confirmed cases, and the wording of (iv) was too restrictive (excluding, for instance, the many studies measuring the duration of symptoms from the date of hospital discharge, rather than from the date of a positive test).

We added two *post hoc* quantitative secondary analyses to reflect the balance of the literature. The first of these was a further evaluation of disease severity. We had found that some studies reported intensive care unit (ICU) versus non-ICU admission, whereas others used the World Health Organisation (WHO) severity scale.^28^ We grouped these studies as follows: ICU admission OR reported as having WHO ‘critical’ or ‘severe’ COVID-19, versus hospitalized patients declared as not admitted to ICU. The second analysis was a broadening of the scope of ‘duration’ to include the 19 studies anchoring on discharge (<12 versus 12+ weeks). We did not combine studies anchoring on the date of PCR testing with those using the date of hospital discharge owing to the wide variability in the duration of COVID-19 hospital admissions. However, *post hoc* we did combine studies using the date of PCR testing with those using the date of onset of symptoms, which we reasoned were often likely to be close together in time. In a *post hoc* qualitative analysis, we inspected scatterplots of reported symptom prevalence against time (separately for: dichotomized <12/12+ weeks, mean duration and median duration of follow-up for all symptoms).

We required a minimum of two studies in each subgroup being compared and a minimum of five eligible studies overall per analysis. Every secondary analysis conducted on each symptom is listed in Supplementary Table 3.

### Data availability

Our full R code can be freely accessed through a repository: https://github.com/katrija/longcovid/blob/main/longcovid_metacode.R

## Results

### Study selection

**Figure 1:**
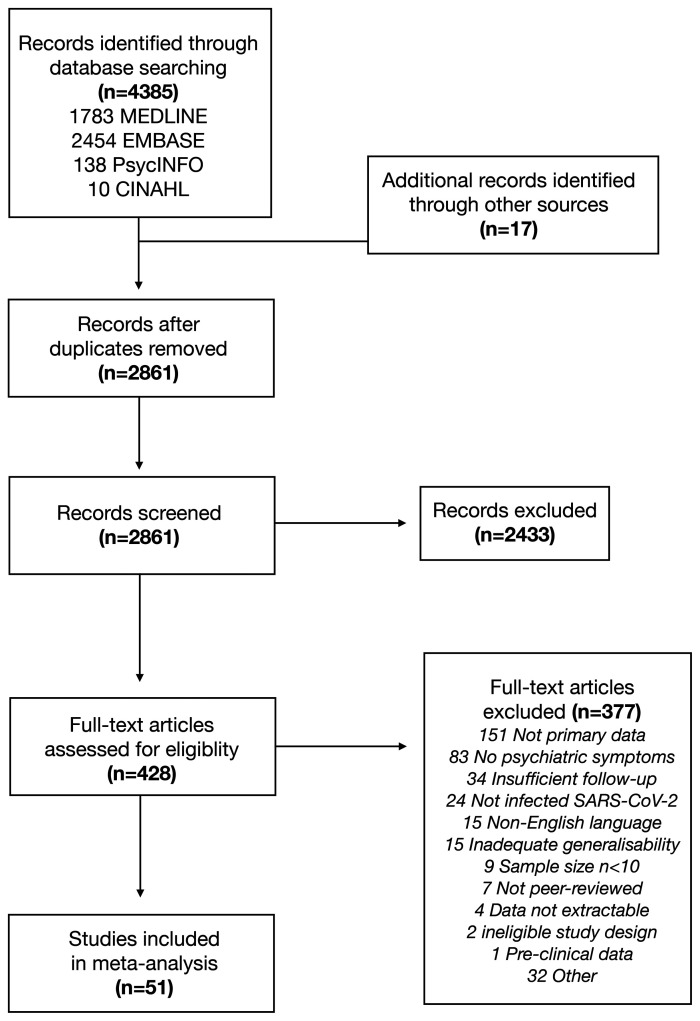
**PRISMA flowchart**.

The search yielded 4385 studies. After de-duplication, we screened the titles and abstracts of 2861 studies, the full text of 428 studies and included 51 eligible studies^27,29–78^ (Fig. 1 and Supplementary Table 4). Brief reasons for excluding studies are listed in Supplementary Table 5. We contacted the authors of seven studies which did not report extricable prevalence data; three replied with usable data.

### Population and study characteristics

The 51 included studies reported data on a total population *n *= 18 917 individuals (sample size range 24–5879; median *n *= 134), *n *= 15 786 (83.4%) of whom had COVID-19 confirmed by PCR. The mean age reported was 50.9 years (SD = 9.4). The largest study (*n *= 5879, 31.1% of entire sample) did not specify sex; of the remainder *n *= 6825 (33.2%) of patients was specified male. Most patients (*n *= 9970, 52.8%) were post-hospital discharge, *n *= 2957 (15.7%) had been treated solely in the community and in *n *= 5962 (31.5%), the location of care was unstated. A metric of COVID-19 severity was reported by half (26/51) of studies, but the number of severe cases declared was often small: only *n *= 1245/18 917 (6.6%) of patients in the full sample were specified as having had ICU admission or WHO critical/severe COVID-19. Ethnicity was reported for only *n *= 2378/18 917 (12.6%) patients, in whom *n *= 1172 (49.3% of specified) were White. Most studies originated in China (13 studies) followed by Italy (six studies), the USA (five studies) and the UK (five studies) (Supplementary Fig. S4 and Table 1).

**Table 1:** Characteristics of included studies

Population characteristics (*n* = # of patients)		
Sample size *(range, median)*	*n*	%
	24–5879, 134	–
COVID-19 confirmed by PCR	15 786/18 917	83.4
Age *(mean, SD)*	50.9, 9.4	–
Sex		
Male	6825	33.2
Female	6213	32.8
Unstated	5879	31.1
Location of COVID-19 treatment		
Hospital	9970	52.7
Community	2957	15.7
Mixed or unstated	5990	31.5
COVID-19 severity		
ICU/WHO critical-sev	1245	6.6
Hospitalized unspecified	8725	46.1
Community only	2957	15.6
Mixed or unstated	5990	31.7
Ethnicity		
White	1172	49.3
Asian	210	8.8
Black	182	7.7
Mixed/multiple	8	0.3
Hispanic	272	11.4
Other (Arab or other)	534	22.5
Unstated	16 539	87.4
Study characteristics (*n* = # of studies)		
Study design	*n*	%
Cohort	43/51	84.3
Cross-sectional	8/51	15.7
Has control group	3/51	5.9
Follow-up duration *(range, mean)*	14-182, 77	–
COVID-19 time-point		
<12 weeks	30/51	58.8
12+ weeks	19/51	37.3
Unstated	2/51	3.9
Definition of follow-up duration		
Post-discharge	23/51	45.1
Post-PCR and post-symptom onset	16/51	31.4
Other	11/51	21.6
Unspecified	2/51	3.9

Most studies (43/51) had a cohort design. Only 2 out of 51 reported symptoms in a control group. The mean duration of follow-up was 77 days (range 14–182 days). Most studies (30/51) examined patients at <12 weeks with 19 out of 51 examining a time-point at 12+ weeks. There was little consensus on the anchor point for calculating follow-up duration: 23 studies reported duration since hospital discharge, 16 studies reported duration post-symptom onset or post-PCR testing, 11 used another definition (e.g. date of virological clearance) and 2 did not specify the anchor point. Study quality assessed using the Newcastle–Ottawa Scale was low in 7 studies, medium in 39 and high in 5 (Supplementary Fig. S1 and Table S6).

### Prevalence of neuropsychiatric symptoms

**Figure 2:**
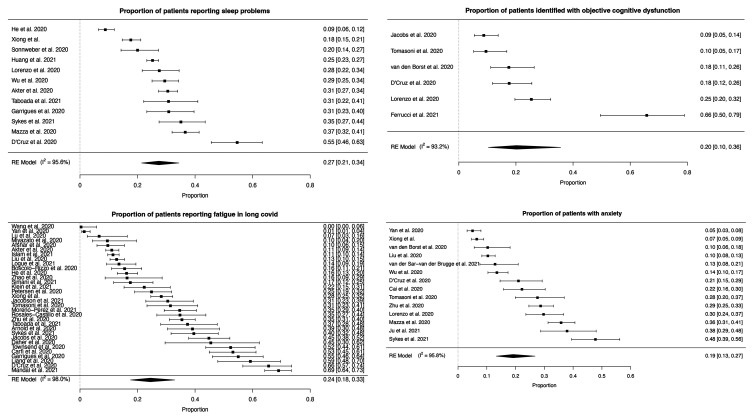
**Forest plots for individual neuropsychiatric symptoms (1–4)**. Sleep problems, fatigue, objective cognitive dysfunction and anxiety. Symptoms are plotted individually. The point prevalence for individual studies is presented with 95% confidence intervals on the right-hand side of each plot. The pooled prevalence and 95% confidence interval for that symptom is shown at the bottom of each plot.

**Figure 3:**
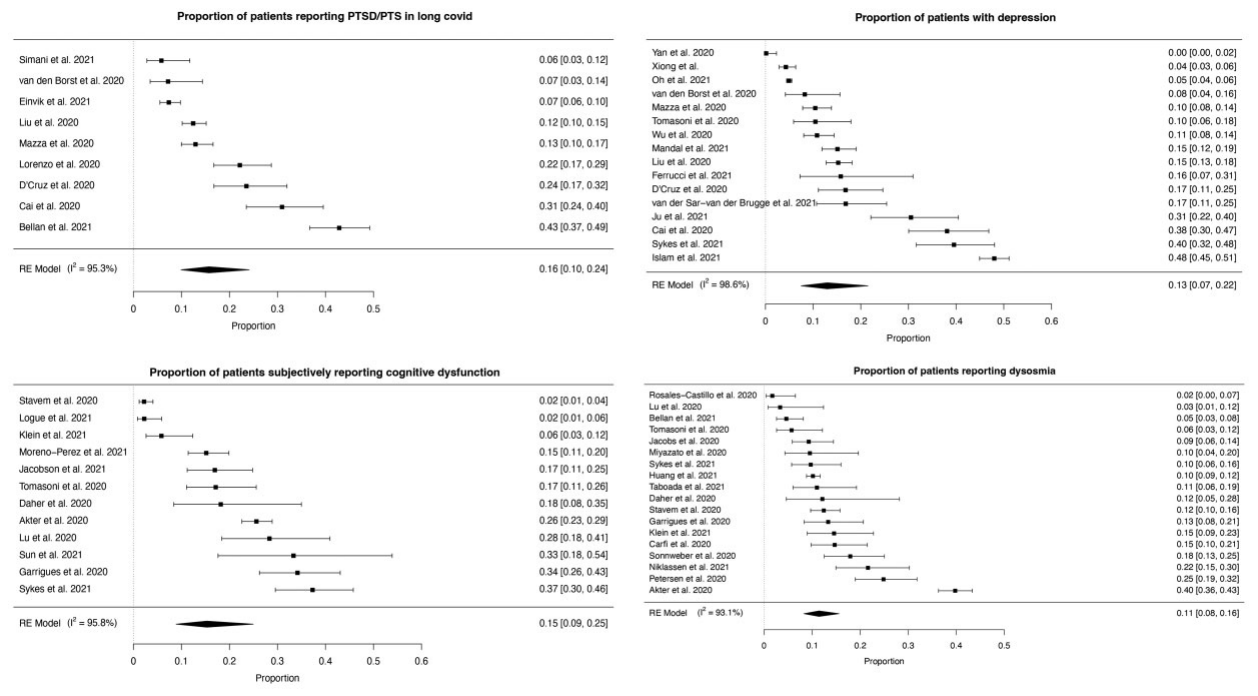
**Forest plots for individual neuropsychiatric symptoms (5–8)**. PTSD/PTS, subjective cognitive dysfunction, depression and dysosmia.

**Figure 4:**
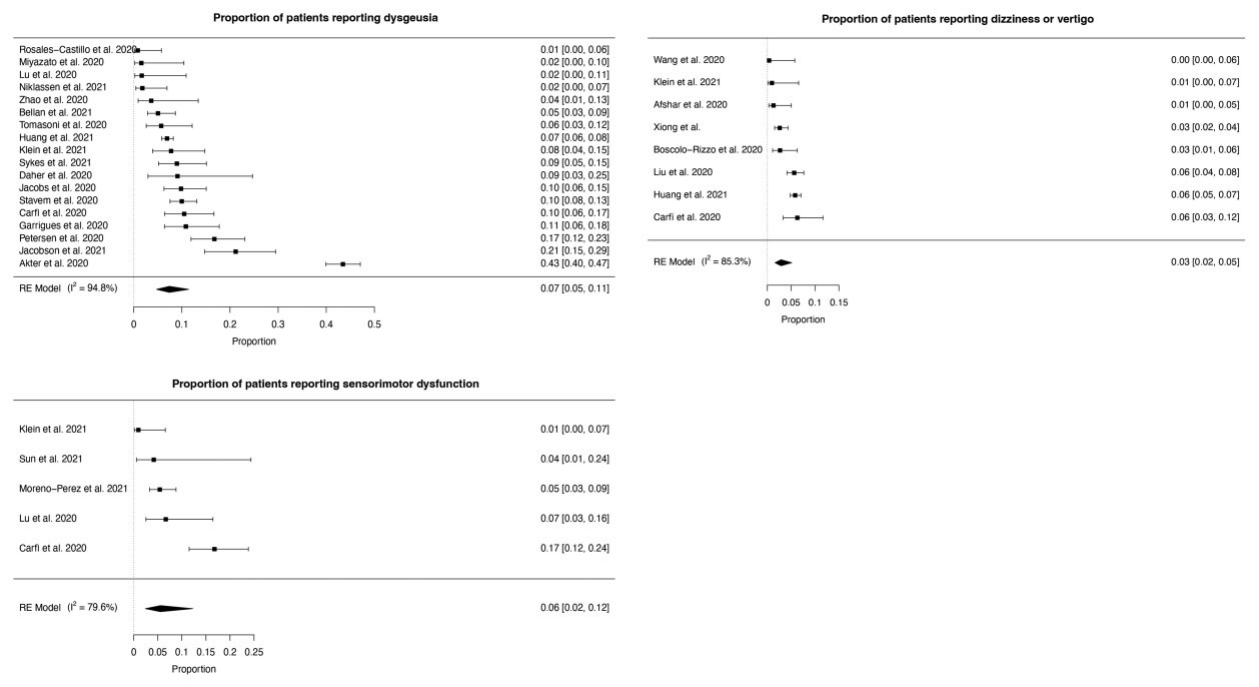
**Forest plots for individual neuropsychiatric symptoms (9–11)**. Dysgeusia, sensorimotor dysfunction and dizziness or vertigo.

The most frequent neuropsychiatric symptom was sleep disturbance [pooled prevalence = 27.4% (95% CI 21.4–34.4%)], followed by fatigue [24.4% (17.5–32.9%)], objectively measured cognitive impairment [20.2% (10.3–35.7%)], anxiety [19.1% (13.3–26.8%)] and post-traumatic stress [15.7% (9.9–24.1%)] (Figs 2–4 and Table 2). More classically ‘neurological’ symptoms such as dysgeusia, headache, sensorimotor disturbance and dizziness/vertigo were less frequent but present in non-negligible amounts (pooled prevalence <10% for each). Speech and language symptoms, panic attacks, mania and hallucinations could not be meta-analysed due to the absence of studies. Heterogeneity was high (*I*^2 ^= 79.6–98.6%, Table 2). The results of the sensitivity analysis were in general similar to the results of the main analysis in terms of the point estimate of prevalence, confidence interval boundaries and heterogeneity (Supplementary Table 7).

**Table 2:** Pooled prevalence of individual neuropsychiatric symptoms

Symptom	*N* studies	*N* subjects	Pooled prevalence	95% CI	*I* ^2^ (%)
Fatigue	32	7501	0.244	0.175, 0.329	98.0
Dysosmia	18	4738	0.114	0.082, 0.156	93.1
Dysgeusia	18	4675	0.074	0.047, 0.114	94.8
Depression	16	10 402	0.129	0.075, 0.215	98.6
Headache	15	4023	0.066	0.036, 0.12	95.2
Anxiety	14	3716	0.191	0.133, 0.268	95.8
Sleep problems	12	4991	0.274	0.214, 0.344	95.6
Subj. cog. dysf.	12	2336	0.153	0.089, 0.25	95.8
PTSD/PTSS	9	2545	0.157	0.099, 0.241	95.3
Dizziness	8	3665	0.029	0.016, 0.051	85.3
Obj. cog. dysf.	6	727	0.202	0.103, 0.357	93.2
Sensorimotor	5	607	0.055	0.024, 0.123	79.6

Symptoms are ranked according to the number of studies reporting them.

Only two studies reported symptoms in control groups, both drawn from healthy populations and neither formally matched to the respective COVID-19 groups.^36,51^ Each reported higher frequencies of sleep disorder, fatigue, dizziness, depression, anxiety and/or psychosis in COVID-19 survivors compared to controls (Supplementary Table 8).

### Secondary analyses

**Figure 5:**
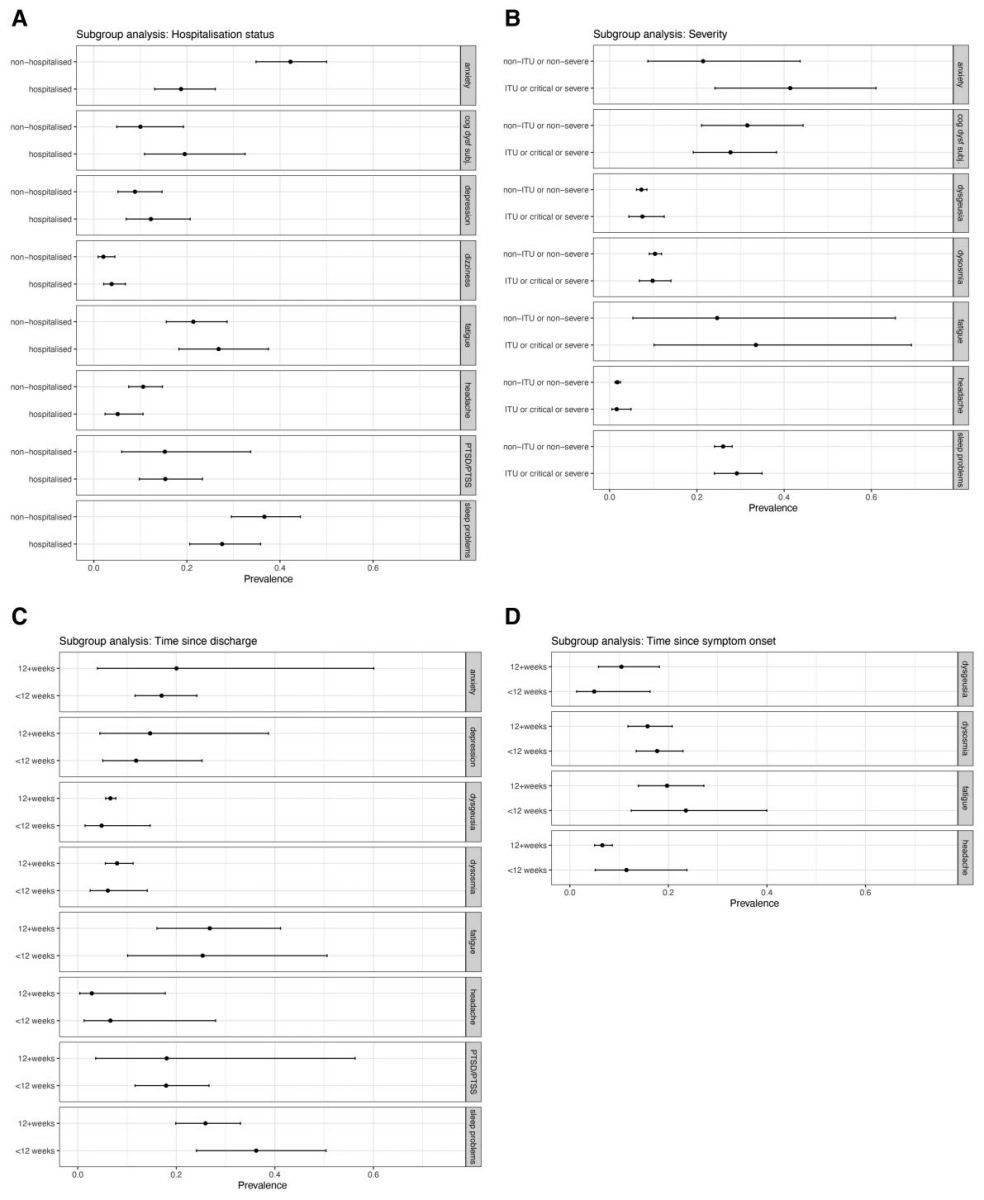
**Pooled symptom prevalence by subgroups**. Four subgroup analyses are shown (major panels **A–D**). Within each analysis, symptoms which were eligible for analysis are plotted individually (identified in the right-hand tab on each minor panel). (**A**) Comparison of pooled prevalence for studies reporting non-hospitalized versus hospitalized samples. (**B**) Comparison of studies reporting patients who had non-ITU/non-severe versus ITU/critical/severe COVID-19. (**C**) Comparison of studies reporting duration of follow-up shorter than 12 weeks post-hospital discharge, versus those reporting longer follow-up. (**D**) Comparison of studies reporting duration of follow-up shorter than 12 weeks since onset of COVID-19 symptoms, versus those reporting longer follow-up.

With the exception of anxiety, which was reported more frequently in non-hospitalized samples, there was no evidence of a differential prevalence of any symptom among hospitalized versus non-hospitalized samples (eight symptoms eligible to be tested), nor among patients admitted to ICU/having WHO ‘critical’ or ‘severe’ illness versus those not requiring ICU (six symptoms). Similarly, there was no evidence of difference among patients surveyed <12 weeks versus 12+ weeks post-discharge (eight symptoms eligible to be tested), nor the same time-points post-symptom onset/PCR test (four symptoms, Fig. 5 and Table 3). Scatterplots of symptom prevalence reported by individual studies, plotted against duration since COVID-19, are presented in Figs S2 and S3.

**Table 3:** Pooled symptom prevalence by subgroups

Subgroup analysis	Symptom	Subgroup	*N* studies	*N* subjects	Pooled prevalence	95% CI
Hospitalization status	Anxiety	Hospitalized	14	3555	0.187	0.131, 0.261
		Non-hospitalized	2	161	0.422	0.348, 0.500
	Depression	Hospitalized	14	3419	0.123	0.070, 0.207
		Non-hospitalized	2	560	0.088	0.052, 0.147
	Sleep problems	Hospitalized	10	4594	0.276	0.206, 0.358
		Non-hospitalized	2	161	0.366	0.296, 0.444
	Subj cog dysf.	Hospitalized	8	1659	0.195	0.109, 0.325
		Non-hospitalized	2	199	0.100	0.049, 0.193
	Dizziness	Hospitalized	5	3220	0.038	0.021, 0.068
		Non-hospitalized	2	290	0.021	0.009, 0.045
	Headache	Hospitalized	10	3049	0.051	0.024, 0.106
		Non-hospitalized	2	283	0.106	0.075, 0.148
	Fatigue	Hospitalized	23	5112	0.268	0.183, 0.375
		Non-hospitalized	3	386	0.214	0.156, 0.286
	PTSD/PTSS	Hospitalized	9	1926	0.154	0.098, 0.233
		Non-hospitalized	3	619	0.153	0.060, 0.337
Severity	Anxiety	ITU/critical/severe	3	220	0.414	0.241, 0.611
		Non-ITU	3	443	0.214	0.088, 0.437
	Sleep problems	ITU/critical/severe	4	264	0.292	0.24, 0.349
		Non-ITU	3	1814	0.260	0.241, 0.281
	Subj cog dysf.	ITU/critical/severe	3	83	0.277	0.192, 0.383
		Non-ITU	3	271	0.316	0.211, 0.444
	Headache	ITU/critical/severe	2	184	0.016	0.005, 0.049
		Non-ITU	2	1680	0.018	0.013, 0.025
	Fatigue	ITU/critical/severe	6	383	0.335	0.102, 0.692
		Non-ITU	5	625	0.246	0.053, 0.655
	Dysgeusia	ITU/critical/severe	3	173	0.075	0.044, 0.125
		Non-ITU	3	1814	0.073	0.062, 0.086
	Dysosmia	ITU/critical/severe	4	264	0.098	0.068, 0.141
		Non-ITU	3	1814	0.104	0.091, 0.119
Time since discharge	Anxiety	<12 weeks	10	2537	0.170	0.116, 0.242
		12+ weeks	2	672	0.200	0.040, 0.601
	Depression	<12 weeks	9	2304	0.118	0.051, 0.252
		12+ weeks	3	710	0.147	0.045, 0.387
	Sleep problems	<12 weeks	3	674	0.362	0.241, 0.504
		12+ weeks	4	2496	0.259	0.199, 0.330
	Headache	<12 weeks	4	624	0.066	0.013, 0.280
		12+ weeks	4	2086	0.029	0.004, 0.178
	Fatigue	<12 weeks	11	2817	0.254	0.101, 0.506
		12 +weeks	7	1074	0.268	0.161, 0.412
	Dysgeusia	<12 weeks	3	334	0.048	0.015, 0.147
		12+ weeks	5	2220	0.066	0.057, 0.077
	Dysosmia	<12 weeks	3	334	0.061	0.025, 0.141
		12+ weeks	5	2256	0.080	0.056, 0.112
	PTSD/PTSS	<12 weeks	5	1202	0.179	0.116, 0.266
		12+ weeks	2	358	0.180	0.036, 0.563
Time since symptom onset	Headache	<12 weeks	4	571	0.115	0.051, 0.238
		12+ weeks	3	742	0.066	0.050, 0.086
	Fatigue	<12 weeks	5	1015	0.235	0.125, 0.400
		12+ weeks	5	634	0.197	0.139, 0.272
	Dysgeusia	<12 weeks	2	254	0.049	0.014, 0.163
		12+ weeks	5	908	0.105	0.058, 0.182
	Dysosmia	<12 weeks	2	254	0.177	0.135, 0.230
		12+ weeks	5	935	0.158	0.118, 0.208

## Discussion

In this systematic review and meta-analysis, we found that neuropsychiatric symptoms are common and persistent after COVID-19. Sleep disorders and fatigue appear to be especially prevalent and may be experienced by as many as one in four patients. Anxiety and post-traumatic stress symptoms also seem particularly common, and cognitive impairment is often objectively detectable. Sensorimotor disturbances and dizziness or vertigo are less common but present in a non-negligible proportion of patients. The prevalence of these symptoms appears to be relatively stable across different points in the first 6 months, between hospitalized and community samples and among hospitalized patients regardless of COVID-19 severity. There are knowledge gaps in the neuropsychiatric consequences of COVID-19 in patients who did not require hospital admission, the impact of ethnicity and the course and frequency of symptoms in the longer term.

These findings should be interpreted cautiously. Three in five studies in this review reported symptoms within the NICE guideline-suggested threshold of 12 weeks for the post-acute phase.^9^ Relatively few eligible community-based or ICU-admitted samples reported our outcomes of interest, making rounded conclusions about the impact of COVID-19 severity difficult to draw. If, in due course, significant symptomatic differences emerge from data comparing hospitalized and non-hospitalized patients, then there could be a case that the term ‘Long COVID’ is best reserved for patients who were not hospitalized—or that a subspecifier could be useful to denote the severity of initial respiratory and/or other symptoms. Non-hospitalized patients were in the minority in this review (with only 15.7% confirmed as such), reflecting the early research focus on hospitalized patients. However, non-hospitalized patients were the majority (91.6%) in a recent large patient-led survey. In our view, patient perspectives on terminology for this initially patient-driven disorder should be considered equally alongside those of clinicians and researchers.^15^

Most studies tended to report outcomes based on patient self-report rather than structured clinical assessments. Meanwhile, the lack of active control groups meant that the specific contribution of COVID-19 to such neuropsychiatric symptoms remains unknown. It is possible that the breadth and frequency of these symptoms represent the natural trajectory of recovery from a serious viral illness. The extent to which neuropsychiatric symptoms were new-onset, versus relapse of an existing condition, was not reported. We did not capture data on comorbidities to elucidate if certain premorbid conditions could make people more susceptible to symptom persistence. Nor did we formally appraise the eligibility of 15 studies in which there was no English-language article available.

Our pooled data, however, imply frequent neuropsychiatric morbidity among COVID-19 survivors in the post-acute phase. These observations echo a recent large study associating COVID-19 with an increased risk of neuropsychiatric clinical diagnoses in the first 6 months, including first-onset insomnia, mood, anxiety, or psychotic disorders and dementia.^14^ The same study found a higher risk of such disorders after COVID-19 compared to other respiratory tract illnesses, indicating that at least some of the apparent neuropsychiatric burden may be COVID-19-specific. Notably, the trajectory of accrual of new psychiatric diagnoses flattened only slightly in the first 6 months, supporting the hypothesis that neuropsychiatric symptoms persist within this timeframe. Our data also broadly support the aforementioned patient-led survey, of 3762 mainly non-hospitalized COVID-19 patients,^15^ in which fatigue, self-reported cognitive dysfunction and other neuropsychiatric symptoms (e.g. dizziness and sensorimotor symptoms) were highly prevalent. Owing to the self-selected nature of that study population—which was recruited mostly via Long COVID support groups and similar organizations—their data would be ineligible to contribute to generalizable estimates of community prevalence in the current meta-analysis; a characteristic which illustrates the difficulty of finding generalizable community-based samples.^79^

The extent to which neuropsychiatric symptom burden impacts upon clinical services remains to be seen, although structuring Long COVID services to be proactive in case identification and treatment seems sensible. It remains possible that the most common symptoms, such as (in descending order of frequency) insomnia, fatigue, cognitive impairment, anxiety, post-traumatic stress and depression may respond to combinations of pharmacological, rehabilitative (e.g. physical and/or occupational therapy), psychological and other treatments.^80–84^ In some instances, persisting symptoms after COVID-19 may reflect initial direct tissue injury mechanisms (e.g. inflammation) overlapping with other or additional mechanisms (e.g. cognitive) as can be seen in other complex disorders arising after an illness like chronic pain. Multidisciplinary approaches are often appropriate for such disorders,^85^ including combinations of neurology, neurorehabilitation, neuropsychiatry, physiotherapy, occupational therapy and psychological input. Such approaches should be incorporated into the planning for ‘Long COVID’ services.

Our results identify areas for further research. Controlled studies are required to separate out the neuropsychiatric consequences of viral illness in general from those potentially specific to COVID-19 in particular. The impact of ethnicity and COVID-19 severity remains to be clarified. Classical epidemiological approaches may be required to generate representative community-based samples, and longer-term follow-up is required. Emerging prospective, longitudinal and multicentre studies will probe the characteristics and aetiology of persistent neuropsychiatric symptoms in patients with COVID-19.^86^ Future trials meanwhile may examine treatments known to be effective in treating neuropsychiatric symptoms in other populations.

## Conclusion

Neuropsychiatric symptoms are common and persistent after recovery from COVID-19. Sleep problems and fatigue predominate and appear to affect roughly one-quarter of survivors. Cognitive impairment, anxiety, post-traumatic symptoms and depression are also common in the first 6 months. There is as yet little evidence that these persisting symptoms relate to the severity of, or duration since, initial infection. Although more research is needed, these early signals suggest a high burden of neuropsychiatric symptoms among COVID-19 survivors. Multidisciplinary services should be resourced accordingly in the post-COVID era.

## Supplementary Material

fcab297_Supplementary_Data

## Abbreviations

ICU =: intensive care unit
NICE =: National Institute for Health and Clinical Excellence
PCR =: polymerase chain reaction
PTSD =: post-traumatic stress disorder
SARS-CoV-2 =: severe acute respiratory syndrome coronavirus 2
WHO =: World Health Organisation.
