# Supplementary material for: Persistent neuropsychiatric symptoms after COVID-19: a systematic review and meta-analysis
Source: Brain Commun. 2021 Dec 17;4(1):fcab297. doi: 10.1093/braincomms/fcab297 (PMC8833580; doi:10.1093/braincomms/fcab297)
Supplement: fcab297_Supplementary_Data [file fcab297_supplementary_data.zip › 006 supplementary_material_legends.docx]

**SUPPLEMENTARY FIGURE LEGENDS**

**Figure S1. Histogram of study quality scores.** The final Newcastle-Ottawa Scale (NOS) score for each included study is plotted in a histogram (total n=51 studies).

**Figure S2. Scatterplots of symptom prevalence over time (dichotomised at 12 weeks).** Each individual symptom is displayed in a minor panel. The point prevalence of that symptom in each individual study is represented by an individual data point. Studies are grouped according to their duration of follow-up (<12 versus 12+ weeks). The anchor point for follow-up duration (since the onset of symptoms/PCR testing, or since discharge) is indicated by the shape of each data point (see legend). In general there is little evidence of differential prevalence according to duration of follow-up.

**Figure S3. Scatterplots of symptom prevalence over time (continuous).** Qualitative illustration of point prevalence estimates of individual symptoms (each minor panel) in individual studies (each data point). The anchor for follow-up duration is indicated by the shape of each point. Due to the relatively small number of studies for most symptoms, a quantitative analysis (e.g. a regression line) has not been conducted. **A’:** Scatterplots of studies reporting *mean* duration of follow-up. **A’’:** Scatterplots of studies reporting *median* duration of follow-up. In general there is little qualitative evidence of a marked change in the reported point prevalence of symptoms with increasing duration since COVID-19.

**Figure S4. Map of studies.** Most studies originated in China, the USA, the UK, and Italy. Map created with mapchart.net.

**LIST OF SUPPLEMENTARY TABLES**

**Table S1.** List of author contributions.

**Table S2.** Full list of data fields extracted from eligible studies.

**Table S3.** All secondary analyses conducted on each symptom.

**Table S4.** Summary of included studies.

**Table S5.** Rationale for excluding studies

**Table S6.** Ranking of study quality.

**Table S7.** Meta-analysis sensitivity analysis.

**Table S8.** Comparison of studies including control groups.
