# Supplementary material for: Persistent neuropsychiatric symptoms after COVID-19: a systematic review and meta-analysis
Source: Brain Commun. 2021 Dec 17;4(1):fcab297. doi: 10.1093/braincomms/fcab297 (PMC8833580; doi:10.1093/braincomms/fcab297)

Figure S1. Histogram of study quality scores

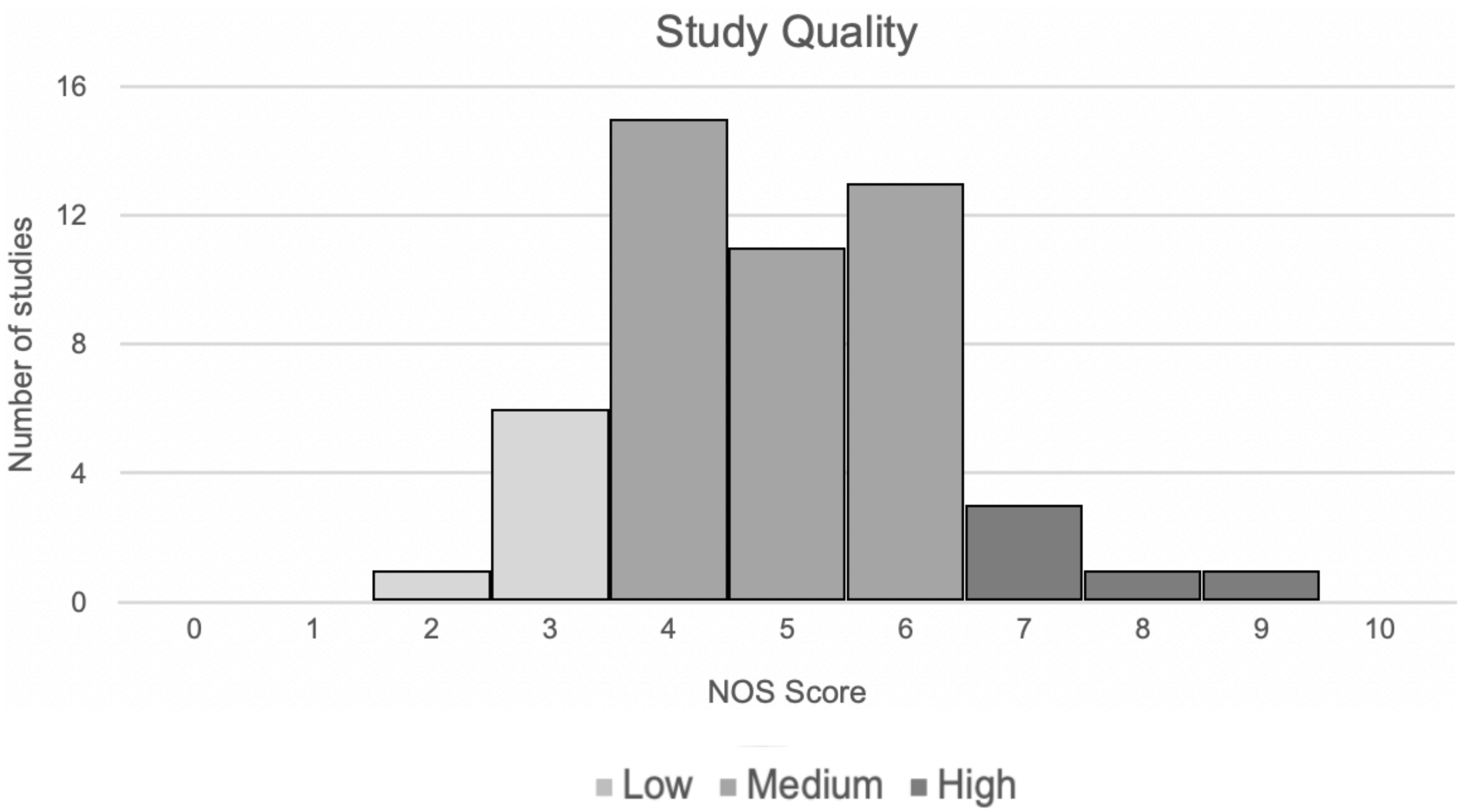

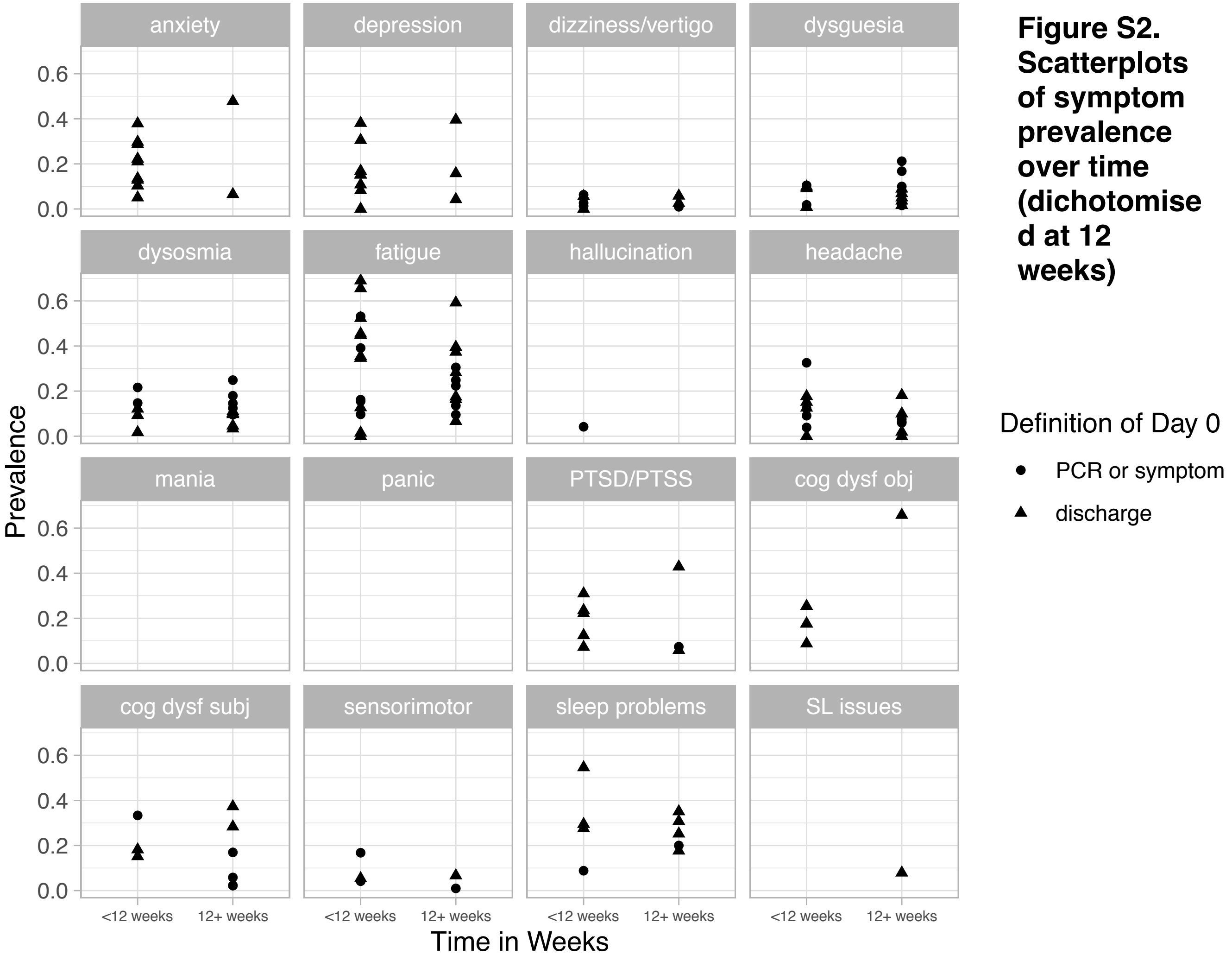

Figure S2. Scatterplots of symptom prevalence over time (dichotomised at 12 weeks)

Figure S3. Scatterplots of symptom prevalence over time (continuous).

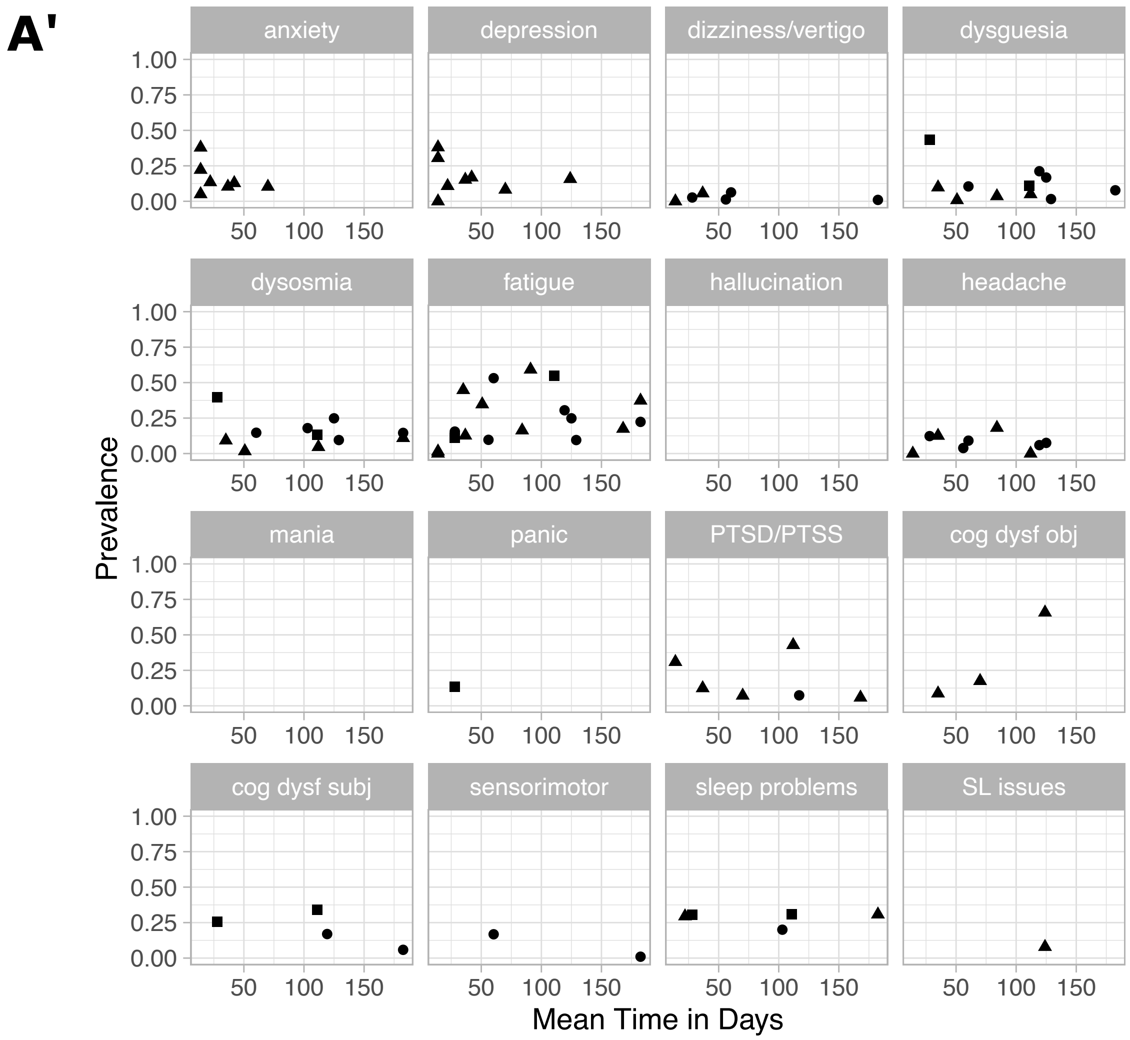

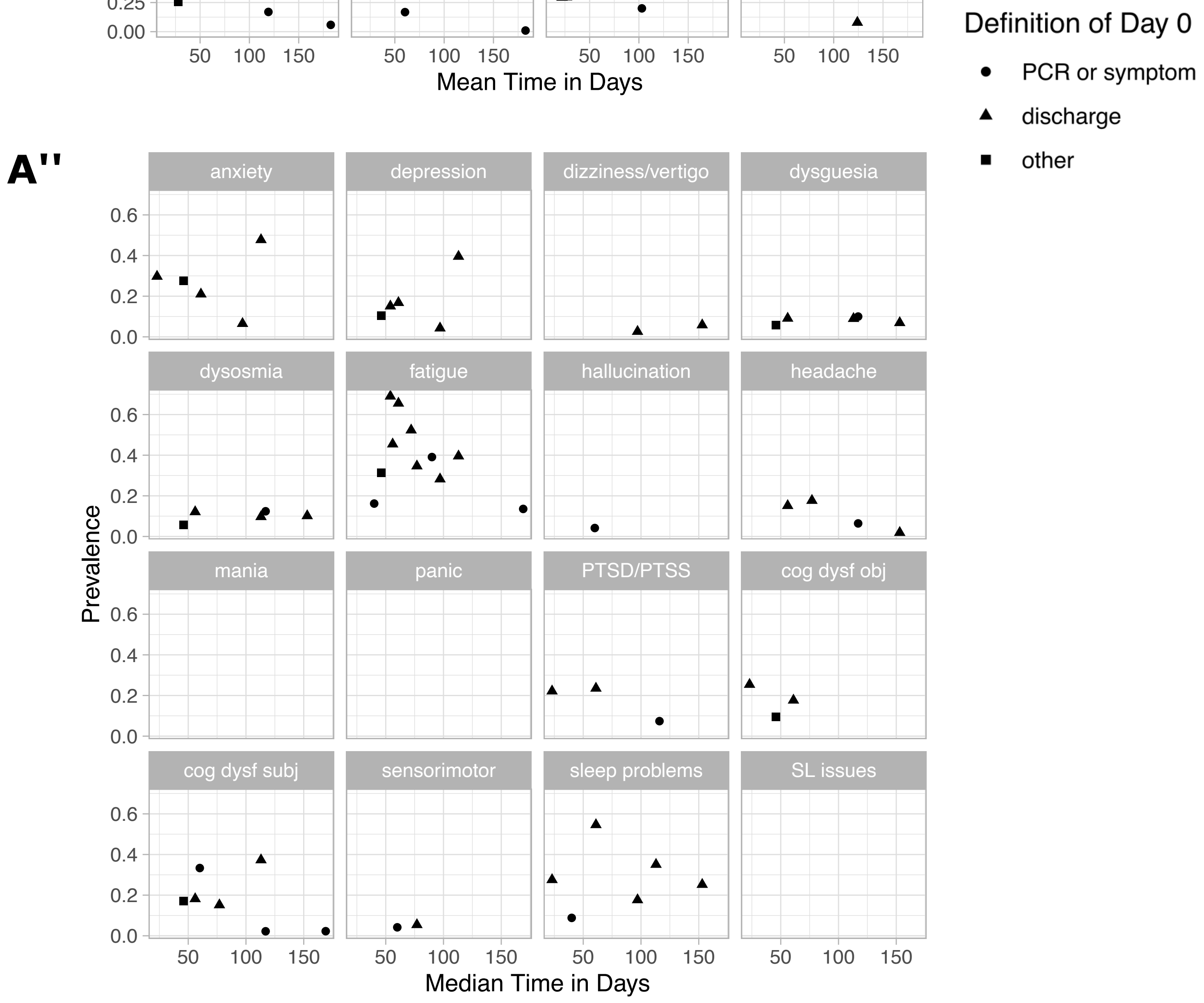

Figure S4. Map of studies

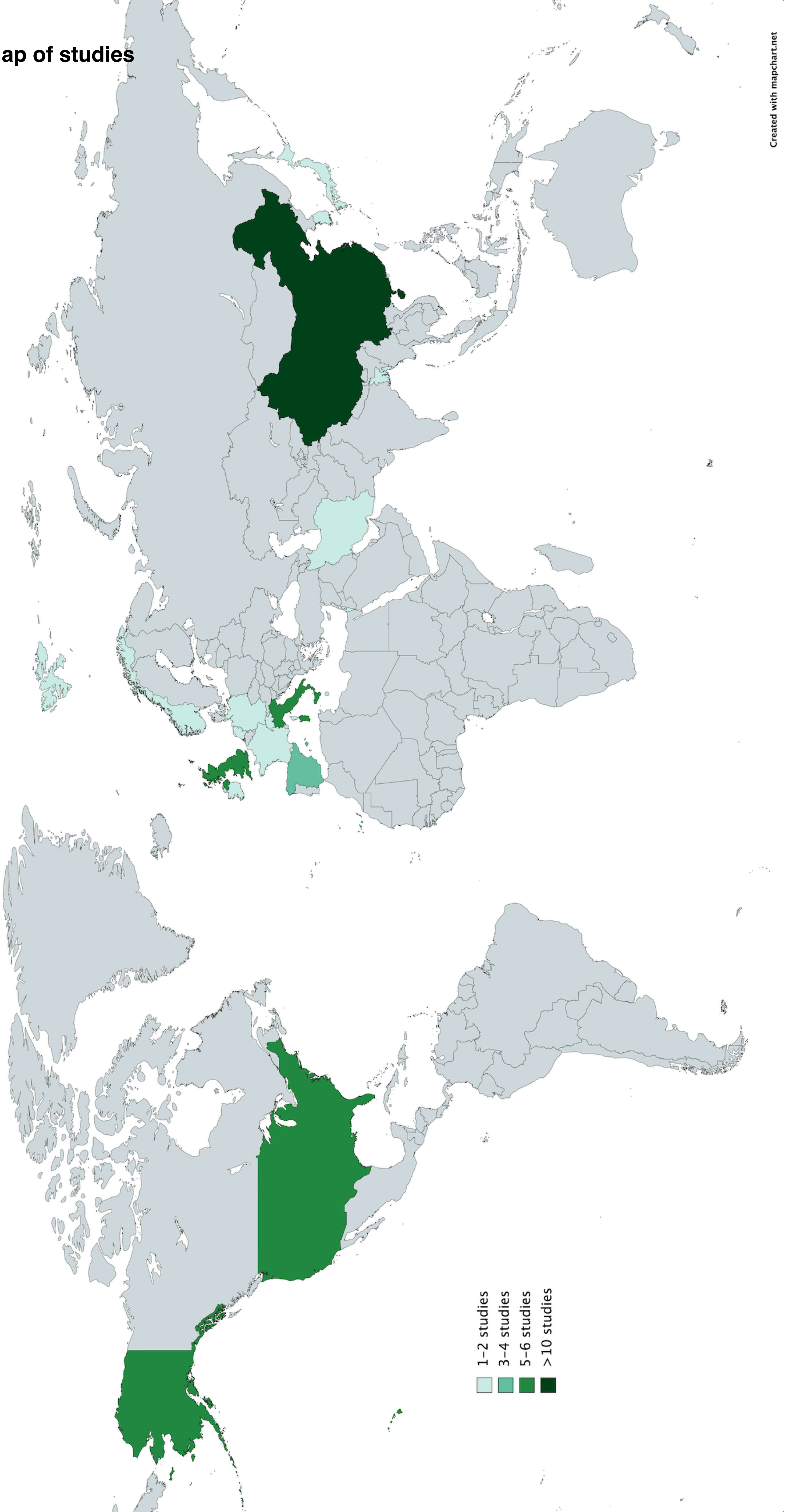

Supplement: fcab297_Supplementary_Data [file fcab297_supplementary_data.zip › 007 Supplementary figures .pdf]
