# Supplementary material for: Persistent neuropsychiatric symptoms after COVID-19: a systematic review and meta-analysis
Source: Brain Commun. 2021 Dec 17;4(1):fcab297. doi: 10.1093/braincomms/fcab297 (PMC8833580; doi:10.1093/braincomms/fcab297)
Supplement: fcab297_Supplementary_Data [file fcab297_supplementary_data.zip › 008 Supplementary tables .docx]

**Table S1.** List of author contributions.

| **Author** | **Contribution(s)** |
| --- | --- |
| *All authors* | - Made a substantial intellectual contribution to the study - Approved the final manuscript |
| Dr James Badenoch | - Conceptualised study - Led and coordinated study - Screened studies for eligibility - Compared to other systematic reviews - Determined meta analysis eligibility - Data extraction methods, extracted data - Checked data extraction - Assisted in cleaning data for analysis - Supported with meta-analysis methods - Assisted in drafting manuscript sections - Arranged funding/COI statements |
| Dr Emma Rachel Rengasamy | - Screened studies for eligibility - Extracted data - Checked data extraction - Supervised quality assessment - Arbitrated quality assessment - Made supplementary table with complete list of studies - Checked adherence to PRISMA guidelines - Adapted to house style |
| Dr Cameron Watson | - Data extraction methods - Supported with meta-analysis methods - Conducted meta-analyses |
| Katrin Jansen | - Supported with meta-analysis methods - Conducted meta-analyses - Assisted with creating tables of results - Created tables of descriptive statistics |
| Miss Stuti Chakraborty | - Screened studies for eligibility - Extracted data - Made supplementary table with complete list of studies - Assisted in writing methods |
| Miss Ritika Dilip Sundaram | - Extracted data - Assisted in cleaning data for analysis - Conducted quality assessment - Made PRISMA flowchart - Created table of excluded studies |
| Mr Danish Hafeez | - Screened studies for eligibility - Extracted data - Data extraction methods- ethnicity - Assisted in cleaning data for analysis - Sorted references |
| Miss Ella Burchill | - Extracted data - Conducted quality assessment - Assisted in writing introduction |
| Mr Aman Saini | - Extracted data - Conducted quality assessment - Made supplementary table with complete list of studies - Created table of extracted data fields |
| Miss Lucretia Thomas | - Screened studies for eligibility - Extracted data - Conducted quality assessment - Made supplementary table with complete list of studies |
| Dr Benjamin Cross | - Screened studies for eligibility - Extracted data - Conducted quality assessment |
| Ms Camille Kaitlyn Hunt | - Screened studies for eligibility - Conducted quality assessment - Made table with characteristics of included studies |
| Miss Isabella Conti | - Extracted data - Conducted quality assessment |
| Ms. Sylvia Ralovska | - Checked extracted data |
| Dr Zain Hussain | - Conducted quality assessment |
| Dr Matthew Butler | - Conceptualised study - Arbitrated quality assessment - Assisted in writing manuscript - Provided senior review of manuscript |
| Dr Thomas Pollak | - Provided senior review of manuscript |
| Dr Ivan Koychev | - Provided senior review of manuscript |
| Dr Benedict Daniel Michael | - Provided senior review of manuscript |
| Prof Dr Heinz Holling | - Provided senior review of manuscript - Supervised application of meta-analytic methods |
| Dr Timothy R Nicholson | - Conceptualised study - Provided senior review of manuscript - Provided senior leadership and advice throughout |
| Dr Jonathan P Rogers | - Conceptualised study - Data extraction methods - Eligibility of studies for data extraction - Provided senior review of manuscript - Provided senior leadership and advice throughout |
| Dr Alasdair G Rooney | - Conceptualised study - Screened studies for eligibility - Consulted on screening studies - Data extraction methods - Extracted data - Checked data extraction - Supported with meta-analysis methods - Created figure of excluded studies - Created tables of descriptive statistics - Wrote first draft of manuscript - Provided senior leadership and advice throughout |

**Table S2.** Full list of data fields extracted from eligible studies.

| **Data field** |
| --- |
| First author of study, year |
| Study populations (specify individually reported subgroups on separate rows) |
| Treatment setting |
| Study design |
| Eligibility for meta-analysis (reason if not) |
| Eligibility for subgroup analysis |
| Need to contact authors with reason |
| Details on data extractions per study |
| Study country/countries of origin |
| Data collection start date in study |
| N infected at long-covid timepoint |
| N infected at long-covid timepoint PCR confirmed |
| N infected at long-covid timepoint not PCR confirmed |
| Number of patients from community |
| Number of patients post-discharge |
| Number of patients reported as 'WHO severe COVID-19' |
| Number of patients reported as 'WHO critical COVID-19' |
| Number of patients admitted to ICU/ITU/intensive care unit |
| Population age (mean) |
| Population age (standard deviation) |
| Population age (median) |
| Population age (IQR Q1) |
| Population age (IQR Q3) |
| Sex (n male) |
| Ethnicity descriptors stated by paper |
| Ethnicity – White |
| Ethnicity – Asian |
| Ethnicity – Black |
| Ethnicity – Mixed/Multiple Ethnic Groups |
| Ethnicity – Hispanic (South American) |
| Ethnicity – Other |
| Long COVID period defined from (post discharge, post PCR test etc.) |
| Mean long COVID duration (days) |
| Standard deviation long-COVID duration (days) |
| Median long-COVID duration (days) |
| IQR Q1 long-COVID duration (days) |
| IQR Q3 long-COVID duration (days) |
| Long-COVID duration categorised (< or > 12 weeks) |
| Definition of control group, if used |
| N control group |
| Control group matched variable(s) |
| **Then we recorded the variables below for each symptom of interest (see footnote).** |
| n infected reported with *x** |
| How *x* was reported in paper* |
| Diagnostic method* |
| Rating scale name with diagnostic threshold (if used) * |
| Mean rating scale score for infected participants (if used) * |
| Standard deviation rating scale score for infected participants (if used) * |
| Median rating scale score for infected participants (if used) * |
| IQR (Q1) rating scale score for infected participants (if used) * |
| IQR (Q3) rating scale score for infected participants (if used) * |
| Mean rating scale score for control participants (if used) * |
| Standard deviation rating scale score for control participants (if used) * |
| Median rating scale score for control participants (if used) * |
| IQR (Q1) rating scale score for control participants (if used) * |
| IQR (Q3) rating scale score for control participants (if used) * |

** Data collected for each of the following neuropsychiatric symptoms: anxiety; depression; mania; panic; hallucinations; sleep changes; objectively reported cognitive dysfunction; subjectively reported cognitive dysfunction; sensorimotor symptoms (such as paraesthesia, numbness, or weakness of specific body parts), dizziness and vertigo; headache; changes in speech or language; and changes in taste or smell; fatigue; and post-traumatic stress disorder or symptoms.*

**Table S3.** All secondary analyses conducted on each symptom.

| **SYMPTOM** | **Included in main meta-analysis?** | ***If 'N', state reason*** | **Included in "hospitalised vs non-hospitalised" analysis?** | ***If 'N',***  ***state reason*** | **Included in "severity" analysis?** | ***If 'N',***  ***state reason*** | **Included in "time" analysis 1: post-discharge <12/ 12+ weeks?** | ***If 'N',***  ***state reason*** | **Included in "time" analysis 2: post-symptom/PCR onset <12/ 12+ weeks?** | ***If 'N',***  ***state reason*** |
| --- | --- | --- | --- | --- | --- | --- | --- | --- | --- | --- |
| Obj cog dysfunction | Y | - | N | <2 non-hosp studies | N | <5 pops | N | <5 pops | N | <5 pops |
| Subj cog dysfunction | Y | - | Y | - | Y | - | N | <5 pops | N | <5 pops |
| Sensorimotor | Y | - | N | <5 populations | N | <5 pops | N | <5 pops | N | <5 pops |
| Dizziness/ vertigo | Y | - | Y | - | N | <5 pops | N | <5 pops | N | <5 pops |
| Sleep | Y | - | Y | - | Y | - | Y | - | N | <5 pops |
| depression | Y | - | Y | - | N | <5 pops | Y | - | N | <5 pops |
| Anxiety | Y | - | Y | - | Y | - | Y | - | N | <5 pops |
| PTSD | Y | - | Y | - | N | <5 pops | Y | - | N | <5 pops |
| Dysguesia | Y | - | N | <2 non-hosp studies | Y | - | Y | - | Y | - |
| Dysosmia | Y | - | N | <2 non-hosp studies | Y | - | Y | - | Y | - |
| Fatigue | Y | - | Y | - | Y | - | Y | - | Y | - |
| Headache | Y | - | Y | - | Y | - | Y | - | Y | - |
| Speech and language | N | <3 studies | - | - | - | - | - | - | - | - |
| Panic | N | <3 studies | - | - | - | - | - | - | - | - |
| Mania | N | <3 studies | - | - | - | - | - | - | - | - |
| Hallucination | N | <3 studies | - | - | - | - | - | - | - | - |
| To be eligible for the main meta-analysis, a symptom had to have been reported by a minimum of three eligible studies. | | | | | | | | | | |
| Eligibility for subgroup analysis required both of the following: i) 2+ studies in each subgroup, and ii) 5+ study populations spread across the two sub-groups.  One study could report hospitalised and non-hospitalised prevalences separately, i.e. contribute two “populations”. | | | | | | | | | | |

**Table S4.** Summary of included studies.

Outcomes refer to all COVID-19 infected patients. Mean values given, where median is used, illustrated by *, IQR/Range specified.

| **Ref** | **Study** | **Setting** | **Population** | **Design** | **N (number of patients with COVID-19)** | **Follow-up duration, mean (sd)** | **Mean age (sd)** | **Male (%), Female (%)** | **Prevalence outcomes (%)** | **Other outcomes reported, mean (sd)** |
| --- | --- | --- | --- | --- | --- | --- | --- | --- | --- | --- |
| [(1)](https://www.zotero.org/google-docs/?af3KGh) | Lorenzo et al· 2020 | Italy | Mixed | Cohort | 185 | Post-discharge 23* (IQR 20-29) | 57* (IQR 48-67)  ‘ | Male 123 (66·5), female 62 (33·5) | Objective cognitive dysfunction 47 (25·4)  Sleep change  51 (27·6)  Anxiety 55 (29·7)  PTSD 41 (22·2) | - |
| [(2)](https://www.zotero.org/google-docs/?aCPH51) | Ju et al· 2020 | China | Hospitalised | Cohort | 95 | Post-discharge  14 (-) | 39* (IQR 30-47) | Male 51 (53·6)  Female 44  (46·3) | Depression 29 (30·5)  Anxiety (37·8) | PHQ-9  2* (Range 0-7)  GAD-7  2* (Range 0-6) |
| [(3)](https://www.zotero.org/google-docs/?a9n9ru) | Simani et al· 2021 | Iran | Hospitalised | Cohort | 120 | Post-discharge 168 (-) | 54·6 (16·9) | Male 80 (66·7)  Female 40 (33·3) | PTSD 7 (5·8)  Fatigue 21 (17·5) | PCL-5  9·3 (10·8)  (-) |
| [(4)](https://www.zotero.org/google-docs/?lo3gKB) | Niklassen et al· 2021 | Italy/Germany | Mixed | Cohort | 111 | Post-PCR test (-) | 44·5 (15) | Male 59 (53·2)  Female 52 (46·8) | Dysguesia 2 (1·8)  Dysnosmia 24 (21·6) | TDI 12·5 (1·5) |
| [(5)](https://www.zotero.org/google-docs/?34GfAv) | Tomasoni et al· 2020 | Italy | Hospitalised | Cross−sectional | 105 | Virological clearance 46* (IQR 43-48) | 55* (IQR 43-65) | Male 77 (73·3)  Female 28 (26·7) | Memory disorder 18 (17·1)  Depression 11 (10·5)  Anxiety 29 (27·6)  Dysguesia 6 (5·7)  Dysnosmia 6 (5·7)  Fatigue 33 (31·4) |  |
| [(6)](https://www.zotero.org/google-docs/?Cb0us7) | Klein et al· 2021 | Israel | Non-hospitalised | Cohort | 103 | Post-PCR test  182·5 (-) | 35 (12) | Male 64 (62·1)  Female 39 (37·8) | Memory disorder 6 (5·8)  Paraesthesia 1 (0·9)  Dizziness/ Vertigo 1 (0·9)  Dysguesia 8 (7·7)  Dysnosmia 15 (14·5)  Fatigue 23 (22·3) |  |
| [(7)](https://www.zotero.org/google-docs/?wvcnZn) | Taboada et al· 2021 | Spain | Hospitalised | Cohort | 91 | Post-discharge 182·5 (-) | 65·5 (10·4) | Male 59 (64·8)  Female 32 (35·1) | Sleep change 28 (30·7)  Dysnosmia 10 (10·9)  Fatigue 34 (37·3)  Anxiety/ Depression 42 (46·1) |  |
| [(8)](https://www.zotero.org/google-docs/?j6H2KS) | Bellan et al· 2021 | Italy | Hospitalised | Cohort | 238 | Post-discharge 112 (-)  PCR confirmed 238  PCR not  confirmed 6  ICU Admission 28 | 61* (IQR 50-71) | Male 142 (59·6)  Female (40·3) | PTSD 102 (42·8)  Dysguesia 12 (5·04)  Dysnosmia 11 (4·6) | IES-R 0 (0) |
| [(9)](https://www.zotero.org/google-docs/?ZMUME6) | Oh et al· 2021 | South Korea | Mixed | Cohort | 5879  Control group n = 93,683 | Other (-) | (-) | (-) | Depression 291 (4·9)  Hallucination 10 (0·2) |  |
| [(10)](https://www.zotero.org/google-docs/?iQHsJK) | Miyazato et al· 2020 | Japan | Hospitalised | Cohort | 63 | Post-symptom onset 129 (21) | 48·1 (18·5) | Male 42 (66·7)  Female 21 (33·3) | Dysgeusia 1 (1·5)  Dysnosmia 6 (9·5)  Fatigue 6 (9·5) |  |
| [(11)](https://www.zotero.org/google-docs/?bxVx6s) | Liang et al· 2020 | China | Hospitalised | Cohort | 76 | Post-discharge 91 (-) | 41·3 (13·8) | Male 21 (27·6)  Female 55 (72·3) | Fatigue 45 (59·2) |  |
| [(12)](https://www.zotero.org/google-docs/?25RL7v) | Sonnweber et al· 2020 | Austria | Mixed | Cohort | 145  Community 36  Hospitalised 109 | Post-PCR test 103 (21) | 57 (14) | Male 82 (56·6)  Female 63 (43·4 ) | Sleep change 29 (20)  Dysnosmia 26 (17·9) |  |
| [(13)](https://www.zotero.org/google-docs/?YfQcxy) | Garrigues et al· 2020 | France | Hospitalised | Cross−sectional | 120 | Other 110·9 (11·1) | 63·2 (15·7) | Male 75 (62·5)  Female 45 (37·5) | Attention 32 (26·6)  Memory 41 (34·1)  Sleep change 37 (30·8)  Dysguesia 13 (10·8)  Dysnosmia 16 (13·3)  Fatigue 66 (55) |  |
| [(14)](https://www.zotero.org/google-docs/?JhNxVy) | Afshar et al· 2020 | USA | Mixed | Cohort | Baseline:594  Week 4: 334  Week 6: 267  Week 8: 155 | (-) (Wk0,4,6,8) | 31·3 (5·1) | Male 0  Female 594 (100) | Week 4: Headache 23 (6·9), Dizziness/fainting 13 (3·9), Fatigue 40 (12·0)  Week 6: Headache 21 (7·9), Dizziness/fainting 3 (1·1), Fatigue 26 (9·7)  Week 8: Headache 6 (3·9), Dizziness/fainting 12 (1·3), Fatigue 15 (9·7) |  |
| [(15)](https://www.zotero.org/google-docs/?FWi2XQ) | He et al· 2020 | China | Hospitalised | Cohort | 420 | Post-symptom onset | 56* (IQR 43-63·8) | Male 207 (49·3)  Female 213 (50·7) | Sleep change 37 (8·7)  Fatigue 68 (16·2) |  |
| [(16)](https://www.zotero.org/google-docs/?M6mICt) | Akter et al· 2020 | Bangladesh | Hospitalised | Cross−sectional | 734 | Virological clearance 28 (-) | (-) | Male 558 (76·0)  Female 176 (24·0) | Loss of concentration 188 (25·6)  Memory loss 141 (19·2)  Sleep change 224 (30·5)  Panic disorder 98 (13·4)  Dysguesia 319 (43·4)  Dysnosmia 292 (39·7)  Fatigue 81 (11·0) |  |
| [(17)](https://www.zotero.org/google-docs/?pN2h8S) | Zhao et al· 2020 | China | Hospitalised | Cohort | 55 | Post-discharge  84 (-) | 47·7 (15·5) | Male 32  (58·2)  Female 23 (41·8) | Headache 10 (18)  Dysguesia 2 (3·6)  Fatigue 9 (16·3) |  |
| [(18)](https://www.zotero.org/google-docs/?6mZdqZ) | D’Cruz et al· 2020 | UK | Hospitalised (ICU only) | Cohort | 119 | Post-discharge  61* (IQR 51-67) | 58·7 (14·4) | Male 74 (62·1)  Female 45 (37·8) | Cognitive Impairment 21 (17·6)  Sleep change 65 (54·6)  Depression 20 (16·8)  Anxiety 25 (21)  PTSD/PTS 28 (23·5)  Fatigue 78 (65·5) | 6-Item Cognitive Impairment Test (>/=8)  PHQ-9 (>9)  GAD-7 (>9)  Trauma Screen Questionnaire (>/=6) |
| [(19)](https://www.zotero.org/google-docs/?iU4Xzc) | Sun et al· 2021 | USA | Mixed | Cohort | 24  Community 17  Hospitalised 7 | Post-symptom onset  60* (IQR 40·8-85) | 45·3 (12·7) | Male 6 (25)  Female 18 (75) | Memory/Cognition 8 (33·3)  Sensorimotor (double vision) 1 (4·2)  Hallucinations 1 (4·2) |  |
| [(20)](https://www.zotero.org/google-docs/?IJmia2) | Islam et al· 2021 | Bangladesh | Mixed | Cross-sectional | 1002  Community 794  Hospitalised 208 | - | 34·7 (13·9) | Male 580 (57·9)  Female 422 (42·1) | Depression 481 (48)  Fatigue/asthenia 115 (11·5) | PHQ-9 9·08 (6·4)  9* |
| [(21)](https://www.zotero.org/google-docs/?ZEzpTS) | Huang et al· 2021 | China | Hospitalised | Cohort | 1733 | Other  186 (-) | 57* (IQR 47-65) | Male 897 (51·8)  Female 836 (48·2) | Headache 33 (1·9)  Dizziness/vertigo 101 (5·8)  Sleep change 437 (25·2)  Dysguesia 120 (6·9)  Dysnosmia 176 (10·2) |  |
| [(22)](https://www.zotero.org/google-docs/?rtYyPk) | Moreno-Perez et al· 2021 | Spain | Mixed | Cohort | 277  Community 95  Hospitalised 182 | Other  77* (IQR 72-85) | 56 (-) | Male 146 (52·7)  Female 131(47·2) | Subjective cognitive dysfunction 42 (15·1)  Sensorimotor (visual loss) 15 (5·4)    Headache 49 (17·7)  Fatigue 96 (34·6) |  |
| [(23)](https://www.zotero.org/google-docs/?L2ffGH) | Halpin et al· 2020 | UK | Hospitalised | Cohort | 100 | Post-discharge 48 (10·3) | - | Male 54 (54)  Female 46 (46) | Concentration/moeory 23 (23),  SLT issues 20 (20),  Fatigue 64 (64),  PTSD 31 (31) |  |
| [(24)](https://www.zotero.org/google-docs/?n33VZV) | Xiong et al | China | Hospitalised | Cohort | 538 | Post-discharge 97* (IQR 95-102) | 52* (41-62) | Male 245 (45·5)  Female 293 (54·4) | Dizziness/vertigo 14 (2·6)  Sleep change 95 (17·3)  Depression 23 (4·3)  Anxiety 35 (6·5)  Fatigue 152 (28·3) |  |
| [(25)](https://www.zotero.org/google-docs/?UcITjY) | Rosales-Castillo et al· 2020 | Spain | Hospitalised | Cohort | 118 | Post discharge 50·8 (6) | 60·2 (15·1) | Male 66 (55·9)  Female 52 (44·1) | Dysgeusia 1 (0·8)    Dysnosmia 2 (1·7)  Fatigue 41 (34·7) |  |
| [(26)](https://www.zotero.org/google-docs/?JCrVnZ) | Jacobs et al· 2020 | USA | Hospitalised | Cohort | 183 | Post discharge 35 (5) | - | Male 112 (61·2)  Female 71 (38·8) | Objective cognitive dysfunction 16 (8·7)  Headache 23 (12·6)  Dysgeusia 18 (9·8)  Dysnosmia 17 (9·3)  Fatigue 82 (44·8) |  |
| [(27)](https://www.zotero.org/google-docs/?xpei8S) | Daher et al· 2020 | Germany | Hospitalised | Cohort | 33 | Post discharge 56* (23) | 64 (3) | Male 22 (66·7)  Female 11 (33·3) | Subjective cognitive dysfunction 6 (18·2)  Headache 5 (15·2)  Anxiety (-)  Depression (-)  Dysgeusia 3 (9·1)  Dysnosmia 4 (12·1)  Fatigue 15 (45·5) | GAD-7 4*(8) |
| [(28)](https://www.zotero.org/google-docs/?fhbgDx) | Arnold et al· 2020 | UK | Hospitalised | Cohort | 110 | Post symptom onset 90* (17) | - | Male 68 (61·8)  Female 42 (38·1) | Fatigue 43 (39·1) |  |
| [(29)](https://www.zotero.org/google-docs/?BlWEim) | Stavem et al· 2020 | Norway | Community | Cross-sectional | 451 | Post symptom onset 117* (162) | 49·8 (15·2) | Male 198 (43·9)  Female 253 (56·1) | Subjective cognitive dysfunction 10 (2·2)  Headache 29 (6·4)  Dysgeusia 45 (10)  Dysnosmia 56 (12·4) |  |
| [(30)](https://www.zotero.org/google-docs/?XiGQ68) | Petersen et al· 2020 | Faroe Islands | Community | Cohort | 173 | Post symptom onset 125 (17) | 39·9 (19·4) | Male 82 (47·4)  Female 91 (52·6) | Headache 13 (7·5)    Dysgeusia 29 (16·8)  Dysnosmia 43 (24·9)  Fatigue 43 (24·9) |  |
| [(31)](https://www.zotero.org/google-docs/?KOTwIi) | Boscolo-Rizzolo· 2020 | Italy | Community | Cohort | 187 | Post PCR test 28 | - | Male 84 (44·9)  Female 103 (55·1) | Headache 19 (23·7)  Dizziness 3 (12·0)  Fatigue 29 (13·9) |  |
| [(32)](https://www.zotero.org/google-docs/?cIxejB) | Van den Borst et al· 2020 | The Netherlands | Mixed | Cohort | 97 | Post discharge 70 (11·9) | - | Male 66 (68)  Female (32) | Depression 8 (8·2)  Anxiety 10 (10·3)  PTSD 7 (7·2) | HADS-depression 15(3·5)  HADS-anxiety 18(4·8)  PCL-5 14·5(15·9) |
| [(33)](https://www.zotero.org/google-docs/?PtCgI9) | Carvalho-Schneider et al· 2021 | France | Mixed | Cohort | 103 | Post symptom onset | - | Male 44 (42·7)  Female 59 (57·3) | Headache 54 (52·4) |  |
| [(34)](https://www.zotero.org/google-docs/?RLvKNY) | Mandal et al· 2021 | UK | Hospitalised | Cross-sectional | 384 | Post discharge 54* (12) | 59·9 (16·1) | Male 238 (62)  Female 146 (38) | Depression 58 (15·1)  Fatigue 265 (69) |  |
| [(35)](https://www.zotero.org/google-docs/?G5PHHe) | Mazza et al· 2020 | Italy | Hospitalised | Cohort | 402 | Other | 57·8 (13·3) | Male 265 (66)  Female 137 (34) | Sleep problems 147 (36·6)  Depression 42 (10·4)    Anxiety 144 (35·8)  PTSD 52 (12·9) | WHIIRS 7·3 (5)  BDI-13 3·3 (4·4)  STAI-state 38·2 (11·1)  PCL 5 14·5 (15·9) |
| [(36)](https://www.zotero.org/google-docs/?guKa6L) | Pellaud et al· 2020 | Switzerland | Hospitalised | Cohort | 196 | Post symptom onset 30 | - | Male 119 (60·7)  Female 77 (39·3) | (-) |  |
| [(37)](https://www.zotero.org/google-docs/?UnEsKu) | Lu et al· 2020 | China | Hospitalised | Cohort | 60 | Post discharge | 44·1 (16) | Male 34 (56·7)  Female 26 (43·4) | Subjective cognitive dysfunction 17 (28·3)  Sensorimotor 4 (6·7)  Headache 6 (10)  Dysgeusia 1 (1·7)  Dysnosmia 2 (3·3)  Fatigue 4 (6·7) |  |
| [(38)](https://www.zotero.org/google-docs/?Q7Ql42) | Zhu et al· 2020 | China | Hospitalised | Cohort | 432 | Post discharge | - | Male 225 (52·1)  Female 207 (47·9) | Anxiety 124 (28·7)  Fatigue 153 (35·4) |  |
| [(39)](https://www.zotero.org/google-docs/?cYAEqZ) | Jacobson et al· 2021 | USA | Mixed | Cohort | 118 | Post PCR test 119·3 (33) | 43·3 (14·4) | Male 63 (53·4)  Female 55 (46·6) | Subjective cognitive dysfunction 20 (16·9)  Headache 7 (5·9)  Dysgeusia 25 (21·2)  Fatigue 36 (30·5) |  |
| [(40)](https://www.zotero.org/google-docs/?c9t5PT) | Skyes et al· 2021 | UK | Hospitalised | Cohort | 134 | Post discharge 113* | 59·6 (14) | Male 88 (65·7)  Female 46 (34·3) | Subjective cognitive dysfunction 50 (37·3)  Sleep problems 47 (35)  Depression 53 (39·6)  Anxiety 64 (47·8)  Dysgeusia 12 (9)  Dysnosmia 13 (9·7)  Fatigue 53 (39·6) |  |
| [(41)](https://www.zotero.org/google-docs/?BKA4kg) | Van der Sar-van der Brugge et al· 2021 | The Netherlands | Hospitalised | Cohort | 101 | Post discharge 42 | 66·4 (12·6) | Male 58 (57·4)  Female 43 (42·6) | Depression 17 (16·8)  Anxiety 13 (12·9) | HADS-depression 3*(5)  HADS-anxiety 4*(5) |
| [(42)](https://www.zotero.org/google-docs/?lNPyJF) | Townsend et al· 2020 | Ireland | Mixed | Cohort | 128 | Other 72* (25) | 49·5 (15) | Male 59 (46·1)  Female 69 (53·9) | Fatigue 67 (52·3) | Chalder Fatigue Scale 15·8 (5·9) |
| [(43)](https://www.zotero.org/google-docs/?HHEG2M) | Wu et al· 2020 | China | Hospitalised | Cross-sectional | 370 | Post discharge 22 | 50·5 (13·1) | Male 203 (54·9)  Female 167 (45·1) | Sleep problems 109 (29·5)  Depression 40 (10·8)  Anxiety 50 (13·5) |  |
| [(44)](https://www.zotero.org/google-docs/?LmkdJQ) | Yan et al· 2020 | China | Hospitalised | Cohort | 337 | Post discharge 14 | - | Male 154 (45·7)  Female 183 (54·3) | Anxiety 17 (5·0)  Fatigue 5 (1·5) |  |
| [(45)](https://www.zotero.org/google-docs/?hhwy0t) | Cai et al· 2020 | China | Hospitalised | Cohort | 126 | Post discharge 14 | 45·7 (14) | Male 60 (47·6)  Female 66 (52·4) | Depression 48 (38·1)  Anxiety 28 (22·2)  PTSD 39 (30·9) | SDS 47·3 (13·1)  SAS 43·2 (10·2)  PTSD-SS 45·5 (18·9) |
| [(46)](https://www.zotero.org/google-docs/?oa2SRs) | Liu et al· 2020 | China | Hospitalised | Cross-sectional | 675 | Post discharge 36·7 | - | Male 317 (47)  Female 358 (53) | Dizziness/vertigo 38 (5·6)  Depression 103 (15·2)  Anxiety 70 (10·3)  PTSD 84 (12·4)  Fatigue 86 (12·7) | PHQ-9 5*(3-8)  GAD-7 4*(2-6)  PCL-5 12*(4-16) |
| [(47)](https://www.zotero.org/google-docs/?D40wws) | Wang et al· 2020 | China | Hospitalised | Cohort | 131 | Post discharge 14 | - | Male 59 (45)  Female 72 (55) | Headache 5 (3·8)    Dizziness/vertigo (0)  Fatigue 7 (5·3) |  |
| [(48)](https://www.zotero.org/google-docs/?F0VKyF) | Carfì et al·2020 | Italy | Hospitalised | Cohort | 143 | Post symptom onset 60·3 (30·6) | 56·5 (14·6) | Male 90 (62·9)  Female 53 (37·1) | Sensorimotor 24 (16·7)  Headache 13 (9·1)  Dizziness/vertigo 9 (6·3)  Dysgeusia 15 (10·5)  Dysnosmia 21 (14·7)  Fatigue 76 (53·1) |  |
| [(49)](https://www.zotero.org/google-docs/?tTbxwi) | Einvik et al· 2021 | Norway | Mixed | Cohort | 583 | Post symptom onset 116* | 51·3 | Male 274 (47)  Female 309 (53) | PTSD 43 (7·4) |  |
| [(50)](https://www.zotero.org/google-docs/?vwMdvD) | Logue et al· 2021 | USA | Mixed | Cohort | 177 | Post symptom onset 169* | 48 (15·2) | Male 76 (42·9)  Female 101 (57·1) | Subjective cognitive dysfunction 4 (2·2)  Fatigue 24 (13·6) |  |
| [(51)](https://www.zotero.org/google-docs/?NetaEH) | Ferrucci et al· 2021 | Italy | Hospitalised | Cohort | 38 | Post-discharge 124(34·2) | 53·5 (12·6) | Male 27 (71·1)  Female 11 (28·9) | Objective cognitive dysfunction 25 (65·7)  Speech and language issues 3 (7·9)  Depression 6 (15·8) | WLG 25·65 (5·23) |

PTSD = Post-traumatic stress disorder , WLG = Word List Generation-WLG, BDI-13= Beck's Depression Inventory (13 item, GAD-7 = Generalized anxiety disorder scale, PCL-5=  DSM-5 PTSD Checklist, SAS = Self-rating anxiety scale , SDS = Self-rating depression scale, PTSD-SS = Post-traumatic stress disorder self-rating scale , PHQ-9=  Patient Health Questionnaire-9, HADS= Hospital Anxiety and Depression Scale, , WHIRS = Women’s Health Initiative Insomnia Rating Scale, STAI-trait = State-Trait Anxiety Inventory , IES-R= The Impact of Event Scale-Revised, PROMIS= Patient-Reported Outcomes Measurement Information System , TDI = threshold, discrimination, and identification, SIT-12= Sniffin' Sticks 12 Identification set , ARTIQ= Acute Respiratory Tract Infection Questionnaire, CFQ = Cognitive failures questionnaire, SLT= Speech or language issues,

[1. De Lorenzo R, Conte C, Lanzani C, Benedetti F, Roveri L, Mazza MG, et al. Residual clinical damage after COVID-19: A retrospective and prospective observational cohort study. Adrish M, editor. PLOS ONE. 2020 Oct 14;15(10):e0239570.](https://www.zotero.org/google-docs/?UFCR2b)

[2. Ju Y, Chen W, Liu J, Yang A, Shu K, Zhou Y, et al. Effects of centralized isolation vs. home isolation on psychological distress in patients with COVID-19. J Psychosom Res. 2021 Apr;143:110365.](https://www.zotero.org/google-docs/?UFCR2b)

[3. Simani L, Ramezani M, Darazam IA, Sagharichi M, Aalipour MA, Ghorbani F, et al. Prevalence and correlates of chronic fatigue syndrome and post-traumatic stress disorder after the outbreak of the COVID-19. J Neurovirol. 2021 Feb;27(1):154–9.](https://www.zotero.org/google-docs/?UFCR2b)

[4. Niklassen AS, Draf J, Huart C, Hintschich C, Bocksberger S, Trecca EMC, et al. COVID ‐19: Recovery from Chemosensory Dysfunction. A Multicentre study on Smell and Taste. The Laryngoscope. 2021 May;131(5):1095–100.](https://www.zotero.org/google-docs/?UFCR2b)

[5. Tomasoni D, Bai F, Castoldi R, Barbanotti D, Falcinella C, Mulè G, et al. Anxiety and depression symptoms after virological clearance of COVID‐19: A cross‐sectional study in Milan, Italy. J Med Virol. 2021 Feb;93(2):1175–9.](https://www.zotero.org/google-docs/?UFCR2b)

[6. Klein H, Asseo K, Karni N, Benjamini Y, Nir-Paz R, Muszkat M, et al. Onset, duration and unresolved symptoms, including smell and taste changes, in mild COVID-19 infection: a cohort study in Israeli patients. Clin Microbiol Infect Off Publ Eur Soc Clin Microbiol Infect Dis. 2021 Feb 16;](https://www.zotero.org/google-docs/?UFCR2b)

[7. Taboada M, Moreno E, Cariñena A, Rey T, Pita-Romero R, Leal S, et al. Quality of life, functional status, and persistent symptoms after intensive care of COVID-19 patients. Br J Anaesth. 2021 Mar;126(3):e110–3.](https://www.zotero.org/google-docs/?UFCR2b)

[8. Bellan M, Soddu D, Balbo PE, Baricich A, Zeppegno P, Avanzi GC, et al. Respiratory and Psychophysical Sequelae Among Patients With COVID-19 Four Months After Hospital Discharge. JAMA Netw Open. 2021 Jan 27;4(1):e2036142.](https://www.zotero.org/google-docs/?UFCR2b)

[9. Oh TK, Park HY, Song I. Risk of psychological sequelae among coronavirus disease‐2019 survivors: A nationwide cohort study in South Korea. Depress Anxiety. 2021 Feb;38(2):247–54.](https://www.zotero.org/google-docs/?UFCR2b)

[10. Miyazato Y, Morioka S, Tsuzuki S, Akashi M, Osanai Y, Tanaka K, et al. Prolonged and Late-Onset Symptoms of Coronavirus Disease 2019. Open Forum Infect Dis. 2020 Nov 1;7(11):ofaa507.](https://www.zotero.org/google-docs/?UFCR2b)

[11. Liang L, Yang B, Jiang N, Fu W, He X, Zhou Y, et al. Three-Month Follow-Up Study of Survivors of Coronavirus Disease 2019 after Discharge. J Korean Med Sci. 2020;35(47):e418.](https://www.zotero.org/google-docs/?UFCR2b)

[12. Sonnweber T, Sahanic S, Pizzini A, Luger A, Schwabl C, Sonnweber B, et al. Cardiopulmonary recovery after COVID-19 – an observational prospective multi-center trial. Eur Respir J. 2020 Dec 10;2003481.](https://www.zotero.org/google-docs/?UFCR2b)

[13. Garrigues E, Janvier P, Kherabi Y, Le Bot A, Hamon A, Gouze H, et al. Post-discharge persistent symptoms and health-related quality of life after hospitalization for COVID-19. J Infect. 2020 Dec;81(6):e4–6.](https://www.zotero.org/google-docs/?UFCR2b)

[14. Afshar Y, Gaw SL, Flaherman VJ, Chambers BD, Krakow D, Berghella V, et al. Clinical Presentation of Coronavirus Disease 2019 (COVID-19) in Pregnant and Recently Pregnant People. Obstet Gynecol. 2020 Dec;136(6):1117–25.](https://www.zotero.org/google-docs/?UFCR2b)

[15. He S, Tian J, Li X, Zhou Y, Xiao M, Zhang Y, et al. Positive RT-PCR Test Results in 420 Patients Recovered From COVID-19 in Wuhan: An Observational Study. Front Pharmacol. 2020 Oct 7;11:549117.](https://www.zotero.org/google-docs/?UFCR2b)

[16. Akter F, Mannan A, Mehedi HMH, Rob MdA, Ahmed S, Salauddin A, et al. Clinical characteristics and short term outcomes after recovery from COVID-19 in patients with and without diabetes in Bangladesh. Diabetes Metab Syndr Clin Res Rev. 2020 Nov;14(6):2031–8.](https://www.zotero.org/google-docs/?UFCR2b)

[17. Zhao Y, Shang Y, Song W, Li Q, Xie H, Xu Q, et al. Follow-up study of the pulmonary function and related physiological characteristics of COVID-19 survivors three months after recovery. EClinicalMedicine. 2020 Aug;25:100463.](https://www.zotero.org/google-docs/?UFCR2b)

[18. D’Cruz RF, Waller MD, Perrin F, Periselneris J, Norton S, Smith L-J, et al. Chest radiography is a poor predictor of respiratory symptoms and functional impairment in survivors of severe COVID-19 pneumonia. ERJ Open Res. 2021 Jan;7(1):00655–2020.](https://www.zotero.org/google-docs/?UFCR2b)

[19. Sun B, Tang N, Peluso MJ, Iyer NS, Torres L, Donatelli JL, et al. Characterization and Biomarker Analyses of Post-COVID-19 Complications and Neurological Manifestations. Cells. 2021 Feb 13;10(2):386.](https://www.zotero.org/google-docs/?UFCR2b)

[20. Islam MdS, Ferdous MostZ, Islam US, Mosaddek ASMd, Potenza MN, Pardhan S. Treatment, Persistent Symptoms, and Depression in People Infected with COVID-19 in Bangladesh. Int J Environ Res Public Health. 2021 Feb 5;18(4):1453.](https://www.zotero.org/google-docs/?UFCR2b)

[21. Huang C, Huang L, Wang Y, Li X, Ren L, Gu X, et al. 6-month consequences of COVID-19 in patients discharged from hospital: a cohort study. The Lancet. 2021 Jan;397(10270):220–32.](https://www.zotero.org/google-docs/?UFCR2b)

[22. Moreno-Pérez O, Merino E, Leon-Ramirez J-M, Andres M, Ramos JM, Arenas-Jiménez J, et al. Post-acute COVID-19 syndrome. Incidence and risk factors: A Mediterranean cohort study. J Infect. 2021 Mar;82(3):378–83.](https://www.zotero.org/google-docs/?UFCR2b)

[23. Halpin SJ, McIvor C, Whyatt G, Adams A, Harvey O, McLean L, et al. Postdischarge symptoms and rehabilitation needs in survivors of COVID‐19 infection: A cross‐sectional evaluation. J Med Virol. 2021 Feb;93(2):1013–22.](https://www.zotero.org/google-docs/?UFCR2b)

[24. Xiong Q, Xu M, Li J, Liu Y, Zhang J, Xu Y, et al. Clinical sequelae of COVID-19 survivors in Wuhan, China: a single-centre longitudinal study. Clin Microbiol Infect. 2021 Jan;27(1):89–95.](https://www.zotero.org/google-docs/?UFCR2b)

[25. Rosales-Castillo A, García de los Ríos C, Mediavilla García JD. Persistencia de manifestaciones clínicas tras la infección COVID-19: importancia del seguimiento. Med Clínica. 2021 Jan;156(1):35–6.](https://www.zotero.org/google-docs/?UFCR2b)

[26. Jacobs LG, Gourna Paleoudis E, Lesky-Di Bari D, Nyirenda T, Friedman T, Gupta A, et al. Persistence of symptoms and quality of life at 35 days after hospitalization for COVID-19 infection. Madeddu G, editor. PLOS ONE. 2020 Dec 11;15(12):e0243882.](https://www.zotero.org/google-docs/?UFCR2b)

[27. Daher A, Balfanz P, Cornelissen C, Müller A, Bergs I, Marx N, et al. Follow up of patients with severe coronavirus disease 2019 (COVID-19): Pulmonary and extrapulmonary disease sequelae. Respir Med. 2020 Nov;174:106197.](https://www.zotero.org/google-docs/?UFCR2b)

[28. Arnold DT, Hamilton FW, Milne A, Morley AJ, Viner J, Attwood M, et al. Patient outcomes after hospitalisation with COVID-19 and implications for follow-up: results from a prospective UK cohort. Thorax. 2021 Apr;76(4):399–401.](https://www.zotero.org/google-docs/?UFCR2b)

[29. Stavem K, Ghanima W, Olsen MK, Gilboe HM, Einvik G. Persistent symptoms 1.5–6 months after COVID-19 in non-hospitalised subjects: a population-based cohort study. Thorax. 2021 Apr;76(4):405–7.](https://www.zotero.org/google-docs/?UFCR2b)

[30. Petersen MS, Kristiansen MF, Hanusson KD, Danielsen ME, á Steig B, Gaini S, et al. Long COVID in the Faroe Islands: A Longitudinal Study Among Nonhospitalized Patients. Clin Infect Dis. 2020 Nov 30;ciaa1792.](https://www.zotero.org/google-docs/?UFCR2b)

[31. Boscolo-Rizzo P, Borsetto D, Fabbris C, Spinato G, Frezza D, Menegaldo A, et al. Evolution of Altered Sense of Smell or Taste in Patients With Mildly Symptomatic COVID-19. JAMA Otolaryngol Neck Surg. 2020 Aug 1;146(8):729.](https://www.zotero.org/google-docs/?UFCR2b)

[32. van den Borst B, Peters JB, Brink M, Schoon Y, Bleeker-Rovers CP, Schers H, et al. Comprehensive health assessment three months after recovery from acute COVID-19. Clin Infect Dis. 2020 Nov 21;ciaa1750.](https://www.zotero.org/google-docs/?UFCR2b)

[33. Carvalho-Schneider C, Laurent E, Lemaignen A, Beaufils E, Bourbao-Tournois C, Laribi S, et al. Follow-up of adults with noncritical COVID-19 two months after symptom onset. Clin Microbiol Infect. 2021 Feb;27(2):258–63.](https://www.zotero.org/google-docs/?UFCR2b)

[34. Mandal S, Barnett J, Brill SE, Brown JS, Denneny EK, Hare SS, et al. ‘Long-COVID’: a cross-sectional study of persisting symptoms, biomarker and imaging abnormalities following hospitalisation for COVID-19. Thorax. 2021 Apr;76(4):396–8.](https://www.zotero.org/google-docs/?UFCR2b)

[35. Mazza MG, De Lorenzo R, Conte C, Poletti S, Vai B, Bollettini I, et al. Anxiety and depression in COVID-19 survivors: Role of inflammatory and clinical predictors. Brain Behav Immun. 2020 Oct;89:594–600.](https://www.zotero.org/google-docs/?UFCR2b)

[36. Pellaud C, Grandmaison G, Pham Huu Thien HP, Baumberger M, Carrel G, Ksouri H, et al. Characteristics, comorbidities, 30-day outcome and in-hospital mortality of patients hospitalised with COVID-19 in a Swiss area - a retrospective cohort study. Swiss Med Wkly. 2020 Jul 13;150:w20314.](https://www.zotero.org/google-docs/?UFCR2b)

[37. Lu Y, Li X, Geng D, Mei N, Wu P-Y, Huang C-C, et al. Cerebral Micro-Structural Changes in COVID-19 Patients – An MRI-based 3-month Follow-up Study. EClinicalMedicine. 2020 Aug;25:100484.](https://www.zotero.org/google-docs/?UFCR2b)

[38. Zhu S, Gao Q, Yang L, Yang Y, Xia W, Cai X, et al. Prevalence and risk factors of disability and anxiety in a retrospective cohort of 432 survivors of Coronavirus Disease-2019 (Covid-19) from China. Federici S, editor. PLOS ONE. 2020 Dec 17;15(12):e0243883.](https://www.zotero.org/google-docs/?UFCR2b)

[39. Jacobson KB, Rao M, Bonilla H, Subramanian A, Hack I, Madrigal M, et al. Patients with uncomplicated COVID-19 have long-term persistent symptoms and functional impairment similar to patients with severe COVID-19: a cautionary tale during a global pandemic. Clin Infect Dis Off Publ Infect Dis Soc Am. 2021 Feb 7;](https://www.zotero.org/google-docs/?UFCR2b)

[40. Sykes DL, Holdsworth L, Jawad N, Gunasekera P, Morice AH, Crooks MG. Post-COVID-19 Symptom Burden: What is Long-COVID and How Should We Manage It? Lung. 2021 Feb 11;](https://www.zotero.org/google-docs/?UFCR2b)

[41. van der Sar-van der Brugge S, Talman S, Boonman-de Winter L, de Mol M, Hoefman E, van Etten RW, et al. Pulmonary function and health-related quality of life after COVID-19 pneumonia. Respir Med. 2021 Jan;176:106272.](https://www.zotero.org/google-docs/?UFCR2b)

[42. Townsend L, Dyer AH, Jones K, Dunne J, Mooney A, Gaffney F, et al. Persistent fatigue following SARS-CoV-2 infection is common and independent of severity of initial infection. PloS One. 2020;15(11):e0240784.](https://www.zotero.org/google-docs/?UFCR2b)

[43. Wu C, Hu X, Song J, Yang D, Xu J, Cheng K, et al. Mental health status and related influencing factors of COVID-19 survivors in Wuhan, China. Clin Transl Med. 2020 Jun;10(2):e52.](https://www.zotero.org/google-docs/?UFCR2b)

[44. Yan N, Wang W, Gao Y, Zhou J, Ye J, Xu Z, et al. Medium Term Follow-Up of 337 Patients With Coronavirus Disease 2019 (COVID-19) in a Fangcang Shelter Hospital in Wuhan, China. Front Med. 2020;7:373.](https://www.zotero.org/google-docs/?UFCR2b)

[45. Cai X, Hu X, Ekumi IO, Wang J, An Y, Li Z, et al. Psychological Distress and Its Correlates Among COVID-19 Survivors During Early Convalescence Across Age Groups. Am J Geriatr Psychiatry Off J Am Assoc Geriatr Psychiatry. 2020 Oct;28(10):1030–9.](https://www.zotero.org/google-docs/?UFCR2b)

[46. Liu D, Baumeister RF, Veilleux JC, Chen C, Liu W, Yue Y, et al. Risk factors associated with mental illness in hospital discharged patients infected with COVID-19 in Wuhan, China. Psychiatry Res. 2020 Oct;292:113297.](https://www.zotero.org/google-docs/?UFCR2b)

[47. Wang X, Xu H, Jiang H, Wang L, Lu C, Wei X, et al. Clinical features and outcomes of discharged coronavirus disease 2019 patients: a prospective cohort study. QJM Mon J Assoc Physicians. 2020 Sep 1;113(9):657–65.](https://www.zotero.org/google-docs/?UFCR2b)

[48. Carfì A, Bernabei R, Landi F, Gemelli Against COVID-19 Post-Acute Care Study Group. Persistent Symptoms in Patients After Acute COVID-19. JAMA. 2020 Aug 11;324(6):603–5.](https://www.zotero.org/google-docs/?UFCR2b)

[49. Einvik G, Dammen T, Ghanima W, Heir T, Stavem K. Prevalence and Risk Factors for Post-Traumatic Stress in Hospitalized and Non-Hospitalized COVID-19 Patients. Int J Environ Res Public Health. 2021 Feb 20;18(4).](https://www.zotero.org/google-docs/?UFCR2b)

[50. Logue JK, Franko NM, McCulloch DJ, McDonald D, Magedson A, Wolf CR, et al. Sequelae in Adults at 6 Months After COVID-19 Infection. JAMA Netw Open. 2021 Feb 1;4(2):e210830.](https://www.zotero.org/google-docs/?UFCR2b)

[51. Ferrucci R, Dini M, Groppo E, Rosci C, Reitano MR, Bai F, et al. Long-Lasting Cognitive Abnormalities after COVID-19. Brain Sci. 2021 Feb 13;11(2).](https://www.zotero.org/google-docs/?UFCR2b)

**Table S6.** Ranking of study quality.

| **Study quality** | **n** | **%** |
| --- | --- | --- |
| Low | 7 | 13.7% |
| Medium | 39 | 76.5% |
| High | 5 | 9.8% |
| Total studies | 51 | 100.0% |

| **Design** | **Number of studies** | **%** |
| --- | --- | --- |
| Cohort | 43 | 84.3% |
| Cross−sectional | 8 | 15.7% |
| Grand Total | 51 | 100% |

**Table S7.** Meta-analysis sensitivity analysis.

| **Symptom** | **N studies** | **N subjects** | **Pooled prevalence** | **95% CI** | **I^2^** |
| --- | --- | --- | --- | --- | --- |
| Fatigue | 32 | 7501 | 0·271 | [0·203, 0·345] | 97·9 % |
| Dysosmia | 18 | 4738 | 0·123 | [0·085, 0·166] | 93·2 % |
| Dysgeusia | 18 | 4675 | 0·086 | [0·05, 0·129] | 94·71 % |
| Depression | 16 | 10402 | 0·155 | [0·092, 0·23] | 98·51 % |
| Headache | 15 | 4023 | 0·081 | [0·042, 0·13] | 95·16 % |
| Anxiety | 14 | 3716 | 0·206 | [0·141, 0·279] | 96·25 % |
| Sleep problems | 12 | 4991 | 0·280 | [0·217, 0·348] | 95·79 % |
| Subj· cog· dysf· | 12 | 2336 | 0·174 | [0·103, 0·258] | 95·37 % |
| PTSD/PTSS | 9 | 2545 | 0·170 | [0·099, 0·255] | 96·2 % |
| Dizziness | 8 | 3665 | 0·029 | [0·014, 0·049] | 85·77 % |
| Obj· cog· dysf· | 6 | 727 | 0·217 | [0·089, 0·381] | 95·68 % |
| Sensorimotor | 5 | 607 | 0·061 | [0·017, 0·124] | 82·69 % |

**Table S8.** Comparison of studies including control group.

| **Ref** | **Symptom** | **COVID-19 patients** | **Control group** |
| --- | --- | --- | --- |
| Oh et al· [31] |  | n=5879 | n=93863 |
|  | *Depression* | 291 (4·9%) | 977 (1·0%) |
|  | *Psychosis* | 10 (0·17%) | 34 (0·04%) |
|  |  |  |  |
|  |  | n=538 | n=184 |
| Xiong et al· [46] | *Depression* | 23 (4·3%) | 2 (1·1%) |
|  | *Dizziness* | 14 (2·6%) | 3 (1·6%) |
|  | *Sleep changes* | 95 (17·7%) | 9 (4·9%) |
|  | *Anxiety* | 35 (6·5%) | 3 (1·6%) |
|  | *Fatigue* | 152 (28·3%) | 17 (9·2%) |

**Supplementary Methods.** Search Strategy.

Search strategy on OVID (MEDLINE, EMBASE, and PsycINFO)

1. (post-acute covid* or postacute covid* or post acute covid*).mp.

2. (post covid* adj3 (illness* or syndrome* or symptom*)).mp.

3. (prolonged adj3 covid*).mp.

4. (persistent adj3 covid*).mp.

5. (chronic adj3 covid*).mp.

6. (long covid* or longcovid* or long-covid*).mp.

7. ((long haul* or longhaul* or long-haul*) adj3 covid*).mp.

8. 1 or 2 or 3 or 4 or 5 or 6 or 7 or 8

9. Remove duplicates from 8

10. (chronic adj3 (complication* or infect* or symptom* or syndrome*)).mp.

11. (long haul* or long-haul* or longhaul*).mp.

12. ((long-term or long term or longterm) adj3 (complication* or consequence* or outcome*)).mp.

13. (Persistent adj3 (infecti* or symptom* or syndrome*)).mp.

14. (prolonged adj3 recovery).mp.

15. sequelae.mp.

16. rehabilitat*.mp.

17. 10 or 11 or 12 or 13 or 14 or 15 or 16

18. (Coronavirus or corona virus or coronavirinae or coronaviridae or betacoronavirus or Covid19 or Covid 19 or Covid-19 or nCoV or CoV 2 or CoV2 or CoV-2 or Sarscov2 or SARS-CoV-2 or 2019nCoV).mp.

19. 17 and 18

20. Remove duplicates from 19

21. 9 or 20

22. Remove duplicates from 21

23. Limit 22 to yr=”2020-Current”

Search strategy for CINAHL

(post-acute covid* or postacute covid* or post acute covid*).mp. or (post covid* adj3 or (illness* or syndrome* or symptom*)).mp. or (prolonged adj3 covid*).mp. or (persistent adj3 covid*).mp. or (chronic adj3 covid*).mp. or (long covid* or longcovid* or long-covid*).mp. or ((long haul* or longhaul* or long-haul*) adj3 covid*).mp.

OR

((chronic adj3 (complication* or infect* or symptom* or syndrome*)).mp. or (long haul* or long-haul* or longhaul*).mp. or ((long-term or long term or longterm) adj3 (complication* or consequence* or outcome*)).mp. or (Persistent adj3 (infecti* or symptom* or syndrome*)).mp. or (prolonged adj3 recovery).mp.

sequelae.mp.or rehabilitat*.mp.) AND (Coronavirus or corona virus or coronavirinae or coronaviridae or betacoronavirus or Covid19 or Covid 19 or Covid-19 or nCoV or CoV 2 or CoV2 or CoV-2 or Sarscov2 or SARS-CoV-2 or 2019nCoV).mp.
